# Empowering Tri‐Functional Palladium's Catalytic Activity and Durability in Electrocatalytic Formic Acid Oxidation Reaction via Innovative Self‐Caging and Alloying Strategies

**DOI:** 10.1002/advs.202405725

**Published:** 2024-10-14

**Authors:** Chan‐Woo Lee, Sun Young Jung, Jeong Ho Ryu, Gyeom Seong Jeon, Ashish Gaur, Min Su Cho, Ghulam Ali, Mingony Kim, Kyung Yoon Chung, Arpan Kumar Nayak, Seoyoon Shin, Jiseok Kwon, Taeseup Song, Tae Ho Shin, HyukSu Han

**Affiliations:** ^1^ Energy AI & Computational Science Laboratory Korea Institute of Energy Research (KIER) Daejeon 34129 Republic of Korea; ^2^ Department of Energy Engineering Konkuk University Seoul 05029 Republic of Korea; ^3^ Department of Materials Science and Engineering Korea National University of Transportation Chungju‐si Chungbuk 27469 Republic of Korea; ^4^ Department of Energy Science Sungkyunkwan University Suwon 16419 Republic of Korea; ^5^ U.S.‐Pakistan Center for Advanced Studies in Energy (USPCASE) National University of Sciences and Technology (NUST) H‐12 Islamabad 25000 Pakistan; ^6^ Center for Energy Storage Research Korea Institute of Science and Technology Hwarang‐ro 14‐gil 5, Seongbuk‐gu Seoul 02792 Republic of Korea; ^7^ Division of Energy and Environment Technology KIST School Korea University of Science and Technology Seoul 02792 Republic of Korea; ^8^ Regional Institute of Education National Council of Educational Research and Training (NCERT) Mysore 570006 India; ^9^ Korea Institute of Ceramic Engineering and Technology KICET 101 Soho‐Ro Jinju 52851 Republic of Korea; ^10^ Department of Energy Engineering Hanyang University Seoul 04763 Republic of Korea

**Keywords:** electrocatalyst, formic acidic oxidation reaction, self‐caging, tri‐functionality, palladium alloy

## Abstract

Direct formic acid fuel cells (DFAFCs) stand out for portable electronic devices owing to their ease of handling, abundant fuel availability, and high theoretical open circuit potential. However, the practical application of DFAFCs is hindered by the unsatisfactory performance of electrocatalysts for the sluggish anodic formic acid oxidation reaction (FAOR). Palladium (Pd) based nanomaterials have shown promise for FAOR due to their highly selective reaction mechanism, but maintaining high electrocatalytic durability remains challenging. In this study, a novel Pd‐based electrocatalyst (UiO‐Pd‐E) is reported with exceptional durability and activity for FAOR, which can be attributed to the Pd nanoparticles encapsulated within a carbon framework where concurrent chemical alloying of Pd and Zr occurs. Further, the UiO‐Pd‐E demonstrates noteworthy multifunctionality in various electrochemical reactions including electrocatalytic ethanol oxidation reaction (EOR) and oxygen reduction reaction (ORR) in addition to the FAOR, highlighting its practical potentials.

## Graphical abstract

Due to their extremely selective reaction mechanism, palladium (Pd)‐based nanomaterials are promising electrocatalyst for FAOR, however their electrocatalytic durability is challenging. A new Pd‐based electrocatalyst (UiO‐Pd‐E) is presented with excellent FAOR durability and activity due to Pd nanoparticles encased in a carbon framework where Pd and Zr get chemically alloyed during FAOR.

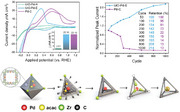

## 1 Introduction

Low‐temperature fuel cells (LTFCs) are attractive future alternatives to conventional fossil fuel‐based power sources and have garnered considerable attention owing to their high conversion rate, ecological energy conversion process, mild working environments, etc.^[^ ^1^, ^2^ ^]^ In particular, DFAFCs are among the most promising LTFC systems for portable electronic devices owing to their ease of handling, abundance of fuels, low membrane permeability, and high theoretical open circuit potential (1.48 V).^[^ ^3^, ^4^, ^5^ ^]^ Additionally, formic acid can be obtained not only from natural biomass but also selectively synthesized from carbon dioxide (CO_2_) via a carbon‐neutral electrosynthetic route using renewable electricity.^[^ ^6^, ^7^, ^8^, ^9^ ^]^ Despite these advantages, the practical application of DFAFCs is hindered by their unsatisfactory electrochemical performance. Particularly in the sluggish anodic FAOR.

The FAOR includes two reaction pathways, that is, direct and indirect dehydration pathways;^[^ ^10^ ^]^ overall reactions are described as HCOOH → CO_2_ + 2(H^+^ + e^−^) and HCOOH → CO* + H_2_O → CO_2_ + 2(H^+^ + e^−^) for the former and later, respectively.^[^ ^11^ ^]^ The direct dehydration pathway occurs through the elementary reactions, 1) HCOOH + * → HCOOH*, 2) HCOOH* → HCOO* + (H^+^ + e^−^), 3) HCOO* → CO_2_ + * +  (H^+^ + e^−^), which is favored at low potentials. In contrast, the indirect dehydration pathways, which proceeds via the following elementary reactions, 1) HCOOH + * → HCOOH*, 2) HCOOH* → COOH* + (H^+^ + e^−^), 3) COOH* + * → CO* + OH*, 4) CO* + OH* → CO_2_ + 2* +  (H^+^ + e^−^).^[^ ^12^ ^]^ Notably, Pd‐based nanomaterials have demonstrated the most promising electrocatalytic FOAR performance owing to their high selectivity for the direct dehydration pathway,^[^ ^13^, ^14^, ^15^ ^]^ which effectively suppresses the catastrophic poisoning of the active sites by CO^*^ and thereby hindering the rapid degradation of catalytic activity. Nonetheless, Pd‐based nanomaterials undergo facile aggregation, which limits their electrocatalytic durability, particularly over prolonged reaction cycles.^[^ ^16^ ^]^ This reduces the active surface area of the catalyst. Numerous efforts have therefore been made to preserve the well‐dispersed catalytic particles and thus maintain excellent electrocatalytic durability. For instance, tuning metal‐support interactions via physicochemical material engineering can anchor catalytic particles onto substrates.^[^ ^17^, ^18^, ^19^ ^]^ Additionally, a number of single‐atom catalysts have been developed to utilize the maximum number of atomically‐dispersed active sites anchored to the matrix to avoid aggregation.^[^ ^20^, ^21^, ^22^ ^]^ Further, the issue of catalyst aggregation and catalytic durability can be overcome using novel exsolution methods, in which catalytic species are evolved from a designed matrix.^[^ ^23^, ^24^, ^25^, ^26^, ^27^ ^]^ However, these strategies require complex and expensive synthetic processes and post‐heat treatments under highly reductive or corrosive conditions, ultimately reducing the economic viability of the catalysts.

In this study, we developed a facile and novel nanomaterial engineering to design a self‐caged and alloyed Pd‐based electrocatalyst exhibiting an exceptional durability and activity for electrocatalytic FAOR. Specifically, a metal‐organic‐framework (MOF) of UiO‐67 (Zr) was employed as a physicochemical precursor to induce the formation of a Pd‐Zr alloy phase in a conductive carbon frame. The frames can physically separate the encaged Pd‐Zr particles within neighboring frames. This physicochemical process spontaneously occurs in situ during electrochemical reactions without any additional chemical or heat treatments. During the self‐caging and alloying processes, Pd‐Zr nanoparticles were caged in MOF‐derived octahedral conductive carbon nanoframes, in which a thin interfacial carbon layer was formed between the cages and caged particles. The durability and electrocatalytic FAOR activity of the resulting self‐caged and alloyed Pd‐Zr catalyst were substantially superior to those of a benchmark commercial Pd/C catalyst (30 wt.% Pd), showing high Pd mass activity. Furthermore, our density functional theory (DFT) calculations predict a significantly favored direct FAOR pathway on the Pd‐Zr catalyst, due to the surface charge dynamics of the Pd‐Zr surface and distinct molecular interactions among Pd and Pd‐Zr surfaces, which originate from the nucleophilic nature of O in HCOO and the electrophilic nature of C in COOH, thus proving beneficial for electrocatalysis. The catalyst also showed promising catalytic activity in the electrocatalytic ORR and EOR reactions in alkaline electrolytes, highlighting its tri‐functional capability and broad catalytic applications.

## 2 Results and Discussion

### 2.1 Transformations of UiO‐67 Framework

**Figure 1:**
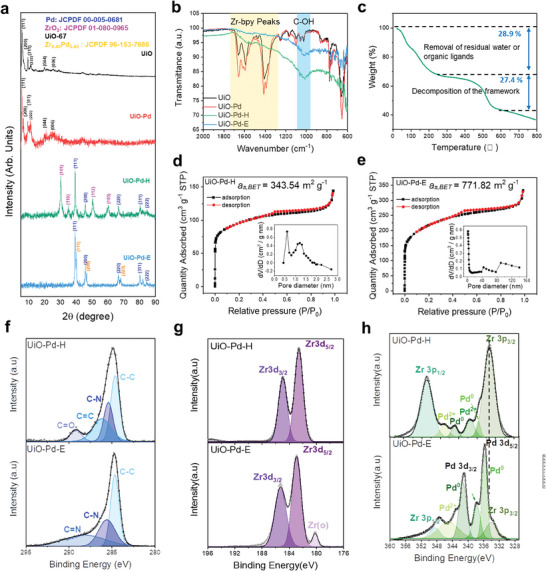
Material characterizations. a) X‐ray diffraction (XRD) patterns of UiO, UiO‐Pd, UiO‐Pd‐H, and UiO‐Pd‐E. b) Fourier transform infrared spectroscopy (FTIR) spectra of UiO, UiO‐Pd, UiO‐Pd‐H, and UiO‐Pd‐E. c) Thermogravimetric analysis (TGA) of UiO under N_2_ atmosphere. Nitrogen adsorption‐desorption isotherm and BJH pore size distribution plots (insets) of d) UiO‐Pd‐H, e) UiO‐Pd‐E. X‐ray photoelectron spectroscopy (XPS) spectra of f) C 1s, g) Zr 3d, and h) Pd 3d for UiO‐Pd‐H and UiO‐Pd‐E.

Pd‐incorporated UiO‐67 (UiO‐Pd) was prepared through a facile one‐step hydrothermal reaction following previous report.^[^ ^28^ ^]^ Subsequent pyrolysis was undertaken at 850 °C to carbonize the frameworks and crystallize Pd compounds (UiO‐Pd‐H). Selective chemical etching of UiO‐Pd‐H in acid (hereafter denoted UiO‐Pd‐E) removed Zr and O atoms. Scanning electron microscopy (SEM) images of the MOF substrate (UiO), UiO‐Pd, UiO‐Pd‐H, and UiO‐Pd‐E (Figure S1, Supporting Information) show that the pristine octahedral structure of UiO was preserved throughout the hydrothermal synthesis, pyrolysis, and etching procedures. The XRD patterns of UiO and UiO‐Pd (**Figure** **1**a) were similar and in good agreement with the theoretical pattern of UiO, demonstrating the successful atomic incorporation of Pd into UiO without the formation of secondary phases. In sharp contrast, the X‐ray diffraction (XRD) pattern of UiO‐Pd‐H reveals a mixed phase consisting of Pd and ZrO_2_, which is likely formed by the carbonization of organic ligands during pyrolysis. The obtained space group for the ZrO_2_ is P4_2_/nmc. Selective chemical etching completely removed the poorly conductive ZrO_2_ phase from UiO‐Pd‐H, as evidenced by the XRD pattern, which was well‐indexed to metallic Pd. The Fourier‐transform infrared (FTIR) spectrum of the UiO‐67 framework showed vibrational bands (1300–1750 cm^−1^) associated with carboxylate linkers.^[^ ^29^ ^]^ These bands disappeared following pyrolysis, and a vibrational band attributed to C‐OH appeared at ≈1050 cm^−1^ (Figure 1b). Further, thermogravimetry differential thermal analysis (TG‐DTA) indicated that the framework decomposed between 400 and 600 °C as evidenced by a sudden reduction in weight (Figure 1c). These results confirm the successful carbonization of the UiO substrate during pyrolysis. Brunauer–Emmett–Teller (BET) adsorption isotherms were obtained to determine the pore structures and BET surface areas of UiO‐Pd‐H and UiO‐Pd‐E (Figure 1d,e). The BET surface area of UiO‐Pd‐E (771.82 m^2^ g^−1^) was more than double that of UiO‐Pd‐H (343.54 m^2^ g^−1^). Importantly, UiO‐Pd‐E exhibits a mesoporous pore structure (Figure 1e inset), whereas UiO‐Pd‐H has a microporous structure (Figure 1d inset). Hence, the selective etching of UiO‐Pd‐E increased the physical surface area by forming mesopores, resulting in an increased number of adsorption sites and facile mass transport, which favors electrochemical reactions.

The surface electronic configurations of UiO‐Pd, UiO‐Pd‐H, and UiO‐Pd‐E were analyzed by X‐ray photoelectron spectroscopy (XPS). The C1s XPS spectra of UiO‐Pd indicates the presence of C─C and C═O bonds (Figure S2, Supporting Information). The C1s spectra of UiO‐Pd‐H shows the presence of C─N and C═C bonds in addition to C─C and C═O bonds, which were likely formed during the carbonization of the framework (Figure 1f, upper panel). In contrast, no C═O bond was observed in the C1s spectra of UiO‐Pd‐E, likely because of the chemical leaching of oxygen (Figure 1f, lower panel). However, the N1s spectra of UiO‐Pd‐E shows the presence of the graphitic, pyrrolic, and pyridinic N (Figure S3, Supporting Information). The Zr3d XPS spectrum of UiO‐Pd‐H shows that the ZrO_2_ phase mainly consists of Zr in the 4^+^ valence state (Figure 1g). In the case of the XPS spectra of the Zr 3d present in UiO‐Pd‐E, we observed the peak for the Zr metal which is generated due to the acid leaching. In addition, the Pd 3d spectrum of UiO‐Pd‐H showed a mixed valence state containing Pd^2+^ and Pd^0^, while that of UiO‐Pd‐H showed a monovalent Pd 2^+^ (Figure S2b, Supporting Information). Notably, positive and negative peak shifts of the major peaks were observed in the Pd 3d spectra of UiO‐P‐E, respectively (lower graphs in Figure 1h), indicating that charge transfer from Pd to Zr occurred during the chemical etching process and thereby demonstrating the feasibility of the formation of the Zr‐Pd alloy phase.

### 2.2 In Situ Formation of Caged Pd‐Zr Alloy Nanoparticles

**Figure 2:**
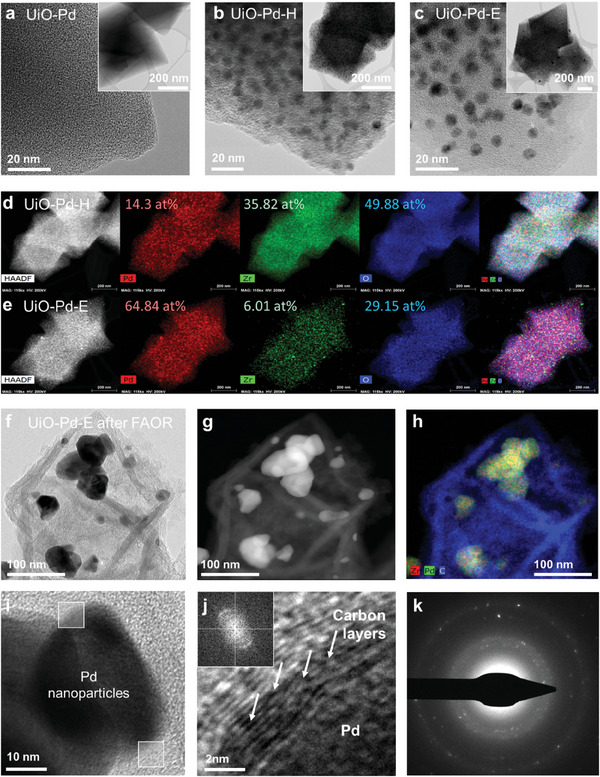
Structural and chemical analysis. Transmission electron microscopy (TEM) and high‐resolution TEM (HR‐TEM) images of a) UiO‐Pd, b) UiO‐Pd‐H, and c) UiO‐Pd‐E. Scanning TEM (STEM) energy dispersive X‐ray spectroscopy (EDX) mapping images for d) UiO‐Pd‐H and e) UiO‐Pd‐E. f,g) STEM image of UiO‐Pd‐E after FAOR and h) corresponding STEM‐EDX mapping image. (i‐j) HR‐TEM images of UiO‐Pd‐E after FAOR and k) collected selected area diffraction (SAED) pattern.

Coordination between the Pd^2+^ precursor (PdCl_2_) with the dipyridyl ligands of the MOF substrate UiO‐67 [Zr_6_O_4_(OH)_4_(bpydc)_6_] afforded UiO‐Pd, which exhibited a well‐defined octahedral shape with a particle size distribution of ≈400–600 nm (**Figure** **2**a). Scanning transmission electron microscopy equipped with energy‐dispersive X‐ray spectroscopy (STEM‐EDX) mapping confirmed the homogeneous distribution of Pd, Zr, and O on the octahedral surfaces of UiO‐Pd (Figure S4, Supporting Information). The well‐distributed Pd formed Pd nanoclusters measuring 1–2 nm on the surface of UiO‐Pd‐H during pyrolysis (Figure 2b). The TEM image of UiO‐Pd‐E showed that these Pd nanoclusters remained intact and were not structurally modified during the acid etching process (Figure 2c). In addition, the STEM‐EDX mapping confirmed the uniform distribution of Pd before and after chemical etching process (Figure 2d,e), and that the distribution of Zr in UiO‐Pd‐E is less dense than that in UiO‐Pd‐H (Figure 2d,e). This is evident in the overlain mapping images (rightmost in Figure 2d,e), quantitative spectra (Figure S5, Supporting Information), and comparative Zr 3d XPS spectra (Figure S6, Supporting Information), indicating the success of the dealloying without structural modulation. Further, the etching was also confirmed using the ICP‐OES analysis of the UiO‐Pd‐H before and after etching (Table. S1, Supporting Information). The wt.% of Pd and Zr in UiO‐Pd‐H is 12.9 and 31.6 respectively, however, after etching the concentration of Zr reduced to 6.08 wt.%. The reduction in the concentration of the Zr after etching process confirms that the selective chemical etching of UiO‐Pd‐H in acid removes Zr and O atoms. In contrast, UiO‐Pd‐E underwent a significant structural change during the FAOR in acidic environments. The hollow structure of the carbon nanoframes initially evolved, as clearly shown in the dark‐field STEM and STEM‐EDX images (Figure 2f–h). FAORs may accelerate the corrosion of the elements inside UiO‐Pd‐E, resulting in hollow octahedral carbon nanoframes. Moreover, the Pd nanoclusters anchored on the octahedral surface of UiO‐Pd‐E aggregated into nanoparticles measuring ≈30–60 nm and were caged in the hollow octahedral frameworks. Importantly, UiO‐Pd‐E also underwent in situ chemical alloying during the FAOR, but no structural changes occurred. To check whether there is further chemical leaching of the Zr is happening during the FAOR we checked the ICP‐OES data before and after FAOR. After 1000 cycles, the quantity of Pd and Zr remains below 0.5 mg (Table S2, Supporting Information), indicating that there is no leaching of Zr occurring in the system during the FAOR process. The caged Pd nanoparticles were alloyed with residual Zr atoms on the octahedral surfaces during the chemical etching process (Figure 2h), which is supported by the XPS spectra (Figure 1g,h). Furthermore, high‐resolution TEM (HR‐TEM) images (Figure 2i,j) and the fast Fourier transform (FFT) pattern (Figure 2j, inset) revealed that thin graphene layers were formed on the surface of the Pd nanoparticles in contact with the carbon cages (Figure 2i,j). In addition, the selective area electron diffraction (SAED) pattern shows diffracted points with a diffusive ring pattern, indicating the coexistence of single‐ and polycrystalline phases (Figure 2k), corresponding to the Pd particles and carbon nanoframes, respectively.

**Figure 3:**
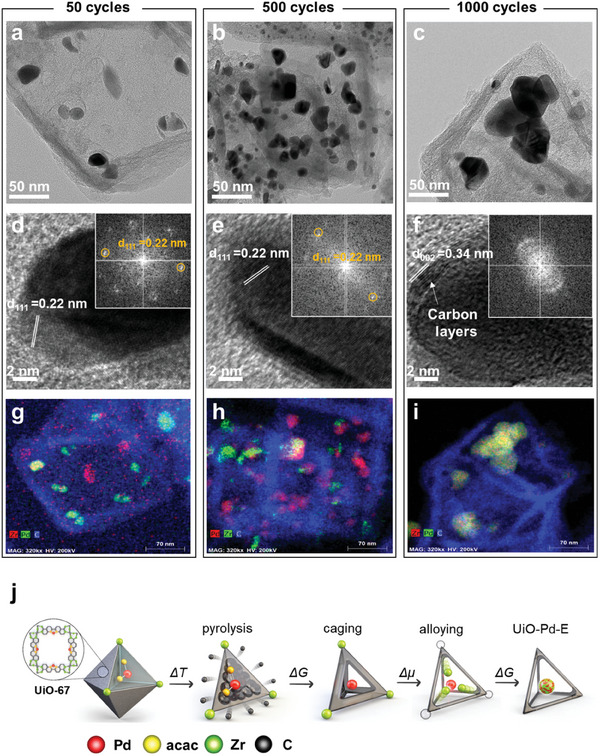
Ex situ TEM studies. STEM images of UiO‐Pd‐E after different FAOR cycles; a) 50, b) 500, and c) 1000, respectively. HR‐TEM images of UiO‐Pd‐E after different FAOR cycles; d) 50, e) 500, and f) 1000, respectively. STEM‐EDX mapping image. The insets show fast‐Fourier transformation patterns of the samples. i–j) HR‐TEM images of UiO‐Pd‐E after different FAOR cycles; g) 50, h) 500, and i) 1000, respectively. j) Schematic illustrations for self‐caging and alloying process in the UiO‐Pd‐E.

These in situ structural and chemical modulations in the UiO‐Pd‐E were further studied in detail through ex situ TEM. The TEM images of the intermediate UiO‐Pd‐E obtained after different FAOR cycles (**Figure** **3**) show that Pd nanoparticles were formed in the hollow octahedral frame over the first 50 FAOR cycles. The number of caged Pd particles increased over the first 500 FAOR cycles, resulting in the formation of aggregates (Figure 3b). At this stage, Pd and Zr were not alloyed, indicating that the elements were physically mixed (Figure 3g,h). Chemical alloying between Pd and Zr was completed after 1000 FAOR cycles (Figure 3c,i), revealing in situ structural and chemical modifications in UiO‐Pd‐E during FAOR. Furthermore, the HR‐TEM images and associated FFT spectra demonstrated the presence of a highly crystalline metallic Pd phase, regardless of the number of FAOR cycles (Figure 3d–f). Thin carbon layers were only observed on the Pd particles in UiO‐Pd‐E after 1000 FAOR cycles (Figure 3f), but were absent after 50 and 500 FAOR cycles (Figure 3d,e), indicating that significant redox reactions are required to create conductive carbon layers at the interface between the Pd and carbon nanoframes. The in situ‐induced thin carbon layers at the interface accelerate charge transfer during electrochemical reactions, thereby improving the electrocatalytic activity of UiO‐Pd‐E. Figure 3j illustrates the in situ nanocaging process in UiO‐Pd‐E during the FAOR. Nanocaging of the Pd clusters occurred during continuous electrochemical FAORs, forming Pd nanoparticles inside the cavity of the MOF substrate. Pristine Pd nanoclusters were alloyed with Zr during the in situ nanocaging process, forming Pd_1‐x_Zr_x_ alloy nanoparticles caged by octahedral frameworks with a thin interfacial carbon layer, significantly increasing the electrocatalytic performance of the catalyst.

### 2.3 Electronic and Local Atomic Configurations of Pd Caged in Carbon Frames

**Figure 4:**
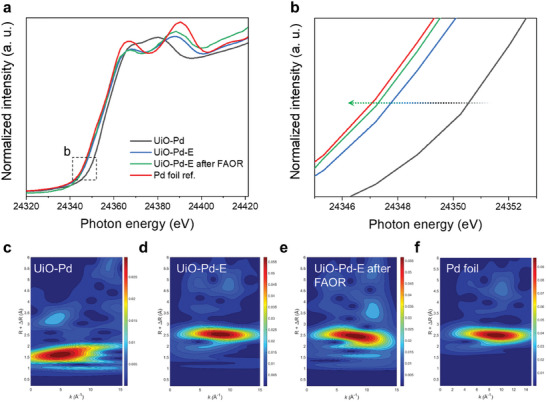
X‐ray absorption spectroscopy. a) X‐ray absorption near edge spectroscopy (XANES) and b) enlarged spectrum of UiO‐Pd, UiO‐Pd‐H, UiO‐Pd‐E, and UiO‐Pd‐E after FAOR. Continuous Cauchy wavelet transform (CCWT) plots of c) UiO‐Pd, d) UiO‐Pd‐E, e) UiO‐Pd‐E after FAOR, and f) Pd‐foil.

X‐ray absorption spectroscopy (XAS) was used to study changes in the local electronic and atomic configurations of UiO‐Pd‐E that occur during the FAOR. **Figure** **4** shows the Pd K‐edge X‐ray absorption near edge spectra (XANES) of UiO‐Pd‐E obtained before and after 1000 FAOR cycles, using metallic Pd foil as a reference. The absorption edge of the Pd K‐edge in the spectrum of UiO‐Pd appeared at a higher photon energy than those in the spectra of the other samples owing to the presence of oxidized Pd in the sample. The Pd K‐edge XANES spectra of UiO‐Pd‐E and metallic Pd foil showed similar oscillations and absorption edges (Figure 4a), indicating that metallic Pd particles were formed in the UiO‐Pd‐E sample. Notably, the absorption edge of the Pd K‐edge was downshifted after the FAOR (Figure 4b), which has been observed in the spectra of alloys of metallic elements.^[^ ^30^ ^]^ This result indicates the chemical alloying of Pd and Zr in the octahedral carbon nanocages during the FAOR, which is consistent with the TEM images and XPS spectra. Additionally, the continuous Cauchy wavelet transformation (CCWT) revealed the local atomic structure of the samples. The CCWT plot of UiO‐Pd shows an intense peak at ≈1.6 Å (Figure 4c), which is indicative of metal‐oxide bonds (Pd‐O). In contrast, the CCWT plot of UiO‐Pd‐E showed an intense peak at ≈2.5 Å (Figure 4d), indicating that the metallic phase dominates in this material. Even after continuous FAOR, an intense peak centered at 2.4 Å was observed in the CCWT plot of UiO‐Pd‐E (Figure 4e), which is similar to that of the Pd foil (Figure 4f), further confirming its robust metallic nature. XANES spectrum of UiO‐Pd‐E at Zr K‐edge is shown in Figure S7a (Supporting Information) along Zr metallic foil, which is noticed at higher energy levels, indicating the presence of some oxidized Zr species. The local structure was further evaluated using EXAFS and CCWT of UiO‐Pd‐E at Zr K‐edge and the phase‐uncorrected *k^3^*‐weighted spectra are shown in Figure S7b (Supporting Information). The results indicate that two small peaks appear before high intense peak where Zr is coordinated with oxygen at 1.637 Å (Zr‐O_a_) which could be associated with the bridging oxygen and at 2.064 Å (Zr‐O_b_) which could be related to the carboxylate ligand in the Pd‐before‐Zr sample. The highest intensity peak is noticed at 2.573 Å which could be associated with the Zr‐M (M = Pd) in the cluster. There was no evidence of the existence of Zr metal as the Zr‐M is noticed at different radial distances compared to the Zr metallic foil. This was further observed in the CCWT where intense peak was noticed at 2.573 Å, indicating formation of Zr‐M in the UiO‐Pd‐E sample (Figure S7c, Supporting Information), where Zr metal shows intense peak at 2.946 Å (Figure S7d, Supporting Information).

### 2.4 Electrocatalytic FAOR Activity and Durability of UiO‐Pd‐E

**Figure 5:**
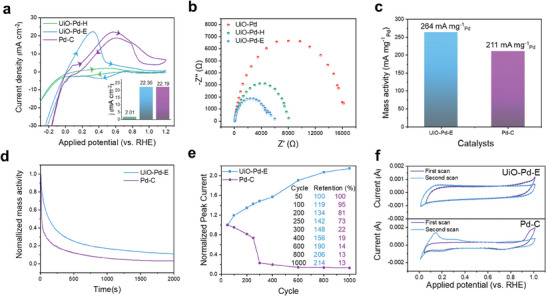
Formic acid oxidation reaction. a) Cyclic voltammetry (CV) curves for UiO‐Pd, UiO‐Pd‐E, and a benchmark Pd/C (30 wt.%) catalysts measured in an electrolyte containing 0.1 m formic acid and 0.1 m HClO_4_ using a scan rate of 10 mV s^−1^. b) Nyquist plots of UiO‐Pd, UiO‐Pd‐H, and UiO‐Pd‐E measured at 0.6 V_RHE_. c) Comparison bar graphs for the Pd mass activity of UiO‐Pd‐E and Pd‐C for FAOR. d) Chronoamperometry measurements at 0.3 V_RHE_ for UiO‐Pd‐E and Pd/C for FAOR. e) Normalized peak currents in different CV curves during 1000 cycles for UiO‐Pd‐E and Pd/C. f) CO striping results for UiO‐Pd‐E and Pd/C.

The electrocatalytic properties of UiO‐Pd‐E in the FAOR were evaluated in a mixed solution of 0.1 m HClO_4_ and 0.1 m HCOOH. A benchmark catalyst (30 wt.% Pd/C) and UiO‐Pd‐H were used as control samples. Cyclic voltammetry (CV) curves were initially obtained over more than 50 cycles at a scan rate of 50 mV s^−1^ in an Ar‐saturated 0.1 m HClO_4_ electrolyte until a steady‐state profile was detected. The CV curve of UiO‐Pd‐E showed a slightly higher peak current density (22.36 mA cm^−2^) to that of the Pd/C catalyst (22.19 mA cm^−2^) (**Figure** **5**a inset), and the peak appeared at a significantly lower potential (0.3 vs 0.6 V_RHE_). This implies that the UiO‐Pd‐E has a much lower overpotential in the FAOR, indicating higher energy conversion efficiency. Additionally, the peak current exhibited by UiO‐Pd‐E in the FAOR was more than ten times greater than that of UiO‐Pd‐H (2.01 mA cm^−2^) (Figure 5a inset). This indicates that the chemical etching step indeed affects the electrocatalytic activity of UiO‐Pd‐E in the FAOR by removing the nonconductive oxygen or zirconium atoms from the frames, thereby improving the charge transfer kinetics. Notably, the charge transfer resistance (*R*_ct_) decreased in the following order UiO‐Pd (≈16000 Ω) > UiO‐Pd‐H (≈8000 Ω) > UiO‐Pd‐E (≈6000 Ω) (Figure 5b). The improved charge transfer in the UiO‐Pd‐E can be also attributed to the formation of the thin interfacial carbon layers between the Pd nanoparticles and carbon frames, as shown in the TEM images (Figure 2j). Importantly, the Pd mass activity in the FAOR, the peak current normalized by Pd content obtained from ICP‐OES (Table S1, Supporting Information), increased more than 25% using the UiO‐Pd‐E (264 mA mg^−1^_Pd_) relative to the benchmark Pd/C (30 wt.%) catalyst (211 mA mg^−1^_Pd_) (Figure 5c), highlighting the superior electrocatalytic mass activity of UiO‐Pd‐E in the FAOR. The electrocatalytic activity of UiO‐Pd‐E in the FAOR was also superior to those of state‐of‐the‐art Pd‐ and Pt‐based electrocatalysts (Table S3, Supporting Information).

Chronoamperometry was performed at 0.3 V_RHE_ to determine the stability of the catalysts, where the UiO‐Pd‐E exhibited higher current retention than the benchmark Pd/C catalyst (Figure 5d). Surprisingly, a continuous FAOR cycle test using UiO‐Pd‐E showed that the peak current gradually increased over 1000 FAOR cycles, reaching more than twice the initial peak current (Figure 5e; FIgure S8a, Supporting Information). This is an unprecedented electrocatalytic behavior for the FAOR. We attributed the enhanced catalytic activity to the in situ physicochemical modifications of UiO‐Pd‐E derived from the electrochemical reaction, that is, the chemical alloying of Pd‐Zr nanoparticles caged in the UiO framework, as shown in the ex situ TEM images (Figure 3). Furthermore, the superior electrocatalytic durability of UiO‐Pd‐E was ascribed to the caged Pd nanoparticles because the carbon frames effectively prevent aggregation of the particles in different frames. The XPS analysis was also carried out after 1000 FAOR cycles and we did not see any major change in the valence state of the Pd and Zr (Figure S9, Supporting Information). After 1000 cycles also we observed the peaks for Zr(0) and Pd(0) in the Zr 3d and Pd 3d XPS spectra. This observation is also confirming the structural robustness of the catalyst towards FAOR. In sharp contrast, the initial peak current abruptly decreased for the benchmark Pd/C catalyst during cycling test, which retained only ≈20% of its electrocatalytic activity after just 300 FAOR cycles (Figure 5e; Figure S8b, Supporting Information), indicating a rapid loss of active sites during electrocatalytic oxidation. This further explains the excellent durability of UiO‐Pd‐E in the FAOR arising from the in situ improvement of the catalytic performance via chemical and structural evolution. In addition, CO‐stripping experiments revealed that the oxidation peak at ≈0.95 V_RHE_ corresponding to CO adsorption onto the catalyst surface was substantially suppressed using UiO‐Pd‐E compared to the Pd/C counterpart (Figure 5f), demonstrating its superior resistance to CO poisoning and indicating the predominance of the direct FOAR pathway.

### 2.5 Theoretical Understanding of FAOR Mechanisms

**Figure 6:**
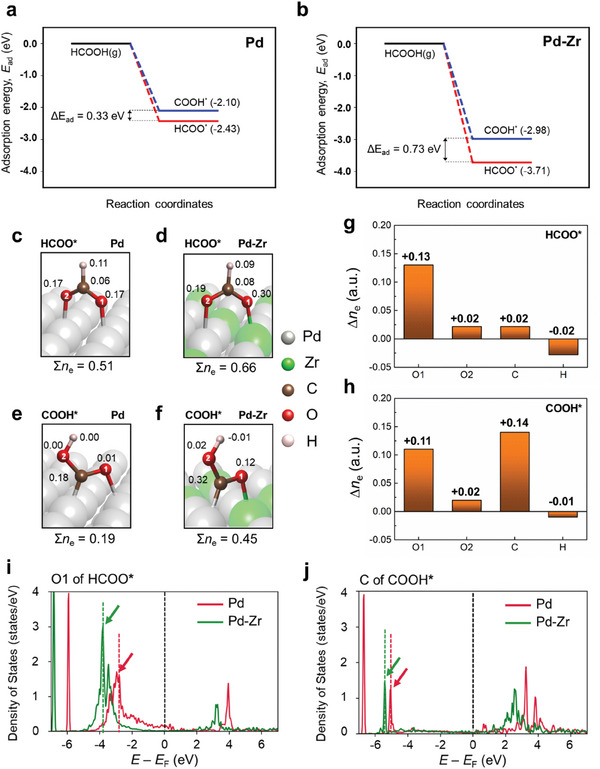
Density functional theory calculations. Adsorption energetics of HCOO and COOH on a) Pd and b) Pd‐Zr surfaces. Stable geometries of adsorbed HCOO (HCOO^*^) and the number of electrons transferred to atoms of HCOO^*^, *n*_e_ (numbers in the boxes) for c) Pd and d) Pd‐Zr surfaces. Stable geometries of adsorbed COOH (COOH^*^) and the number of electrons transferred to atoms of COOH^*^, *n*_e_ (numbers in the boxes) for c) Pd and d) Pd‐Zr surfaces. Difference in electrons transferred to g) HCOO^*^ and h) COOH^*^ on Pd and Pd‐Zr surfaces, ∆*n*_e_. Partial density of states of i) O1 of HCOO^*^ and j) C of COOH^*^ on Pd and Pd‐Zr surfaces.

**Figure** **6**a,b shows the adsorption energies of HCOO and COOH for dehydrogenation via the direct and indirect paths, respectively. To study the effects of the chemical alloying of Pd‐Zr in UiO‐Pd‐E, we compared the adsorption energies of Pd and Pd‐Zr surfaces (Pd:Zr = 3:1, consistent to the experimental results), respectively. Figure S9 in the Supporting Information depicts slab models of Pd (111) and Pr‐Zr (111) surfaces used in the calculations. For both pristine Pd and Pd‐Zr surfaces, HCOO for the direct dehydrogenation shows strong adsorption behavior in contrast to COOH for indirect dehydrogenation. Specifically, energy difference between HCOO and COOH adsorptions [∆*E*_ad_ = *E*_ad_ (COOH) – *E*_ad_ (HCOO)] is 0.73 eV on Pd‐Zr surface which is larger than that on Pd surface (0.33 eV) by 0.40 eV. This increased ∆*E*_ad_ for the Pd‐Zr surface indicates Zr improves dehydrogenation selectivity.

Change of Bader charges by adsorption of HCOO and COOH on Pd‐Zr surface are compared with those on pristine Pd surface (Figure 6c–f). The stabilities of HCOO^*^ and COOH^*^ are primarily dependent upon adsorption‐driven electronic redistribution and corresponding charge dynamics of atoms that undergo dehydrogenation (O1 for HCOO^*^ and C for COOH^*^). Electronic transfer from the surface to adsorbed molecules is always larger in HCOO^*^ in comparison with COOH^*^. Also, Zr in Pd‐Zr surface consistently improves electronic transfer from the surface to the molecules. Specifically, electrons transferred from the surface to HCOO^*^ are 0.51e (Pd) and 0.66e (Pd‐Zr) (Figure 6c,d). Similarly, electrons transferred from the surface to COOH^*^ are 0.19e (Pd) and 0.45e (Pd‐Zr) (Figure 6e,f). Charge redistribution in HCOO^*^ by surface Zr doping (Pd surface → Pd‐Zr surface) is most prominent on O1 that is bonded with Zr ((∆q = +0.13 in Figure 6g). In contrast, for COOH^*^ by the Zr doping, the highest electronic redistribution was observed on C bonding with Pd (∆q = +0.14 in Figure 6h). However, in comparison with HCOO^*^ where electronic redistributions at atoms do not lose H are minimal (e.g., ∆q of O2 = 0.02 in Figure 6g), O1 of COOH^*^ on Zr also shows electronic redistribution comparable to C (∆q of O1 = 0.11 in Figure 6h). This is partly because COOH^*^ does not favor a molecular geometry involving bonding between C (in COOH)^*^ and Zr (in Pd‐Zr surface).

The relatively large increase in *E*_ad_ (HCOO) by Zr is attributed to the disparate electron demand abilities between the hydroxyl O atom (O1) in HCOO^*^ and the C atom in COOH^*^. In formic acid (HCOOH), the hydroxyl O exhibits nucleophilic characteristics while the C atom is electrophilic. The nucleophilic O tends to carry a negative charge, whereas the electrophilic C tends to become positively charged. Notably, dehydrogenation does not alter these inherent features. Even after losing hydrogen, the hydroxyl O1 in HCOO retains its lone pair electrons, rendering it negatively charged (anion), while the C in COOH becomes more positively charged (cation). Considering that the atoms involved in dehydrogenation mainly determine the stabilities of HCOO^*^ and COOH^*^ on Pd‐Zr surface, the interactions between the O and C atoms and surface Zr in Pd‐Zr respectively will provide key insights to understand the improved dehydrogenation selectivity depicted by the increased ∆*E*_ad_ in Figure 6b. Given the lower electronegativity of Zr (1.33) in comparison to the host Pd (2.20), Zr within the Pd‐Zr alloy exhibits a positively charged character, effectively behaving as a cation. Consequently, the interaction between the cationic Zr and the anionic O in HCOO^*^ is anticipated to be more favorable than the interaction between Zr and C in COOH^*^ both of which are cationic. This is why the stable configuration of COOH^*^ on the Pd‐Zr surface is achieved through C‐Pd bond rather than C‐Zr bond.

Origins for the improved electronic transfer due to chemical alloying of Zr and Pd may differ in direct or indirect (i.e., HCOO^*^ and COOH^*^) FAOR pathways. For HCOO^*^, where the O atoms bond with the surface Pd and Zr, respectively, the transferred electrons will be readily redistributed mainly by the nucleophilic O atoms. However, for COOH^*^, where the C atom binds with the surface Pd atom, the redistribution of electrons will not be favored due to the electrophilic nature of the C atom. To confirm this, Figure 6i,j presents the density of states (DOS) plots for the O1 in HCOO^*^ and C in COOH, respectively, on the Pd‐Zr surfaces. It is clearly noted that the DOS plot of O1 for HCOO^*^ shows a significant down‐shift of main peaks (≈−1 eV, Figure 6i), while a relatively small peak shift is observed in the DOS plot of C for HCOO^*^ (Figure 6j). These results support the more favorable charge redistributions at the Pd‐Zr surface for direct FAOR pathway than the indirect pathway, which is related to the enhanced FAOR activity for the Pd‐Zr alloyed phase.

### 2.6 Multi‐Functionality of UiO‐Pd‐E for Diverse Electrocatalytic Reactions

**Figure 7:**
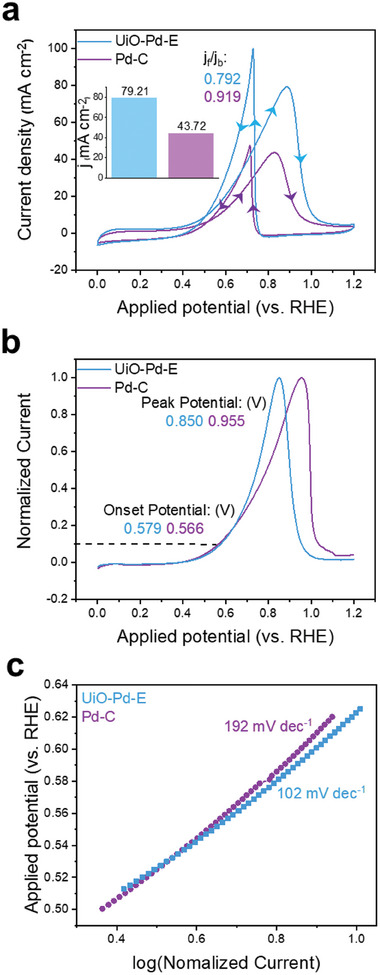
Ethanol oxidation reaction. a) CV and b) linear scanning voltammetry (LSV) polarization curves for UiO‐Pd‐E and a benchmark Pd/C (30 wt.%) catalysts measured in an electrolyte containing 1.0 m KOH and 1.0 m Ethanol using a scan rate of 10 mV s^−1^. c) Corresponding Tafel plots.

In addition to the FAOR, UiO‐Pe‐E is applicable in a number of diverse electrocatalytic reactions. The electrocatalytic activity of UiO‐Pd‐E in the ethanol oxidation reaction (EOR) was also evaluated in an Ar‐saturated 1.0 m KOH electrolyte containing 1.0 m EtOH. The CV curves obtained with UiO‐Pd‐E show higher oxidative currents in both the forward and backward scans relative to those obtained using the benchmark Pd/C catalyst (**Figure** **7**a), revealing the higher catalytic activity of the former in the EOR. During the forward scan, the UiO‐Pd‐E afforded a current density of 79.21 mA cm^−2^, which is ≈1.8 times higher than that of the Pd/C (43.72 mA cm^−2^) (Figure 7a inset). Furthermore, the onset potentials of UiO‐Pd‐E and Pd/C, defined as the potential at which current reaches 10% of the peak current in the linear scanning voltammetry (LSV) curves (Figure 7b), were similar at 0.579 V_RHE_ and 0.566 V_RHE_, respectively. The EOR peak potential was located at a significantly lower potential in the LSV curve of UiO‐Pd‐E (0.85 V_RHE_) than in that of Pd/C (0.955 V_RHE_) (Figure 7b) with a lower Tafel slope (Figure 7c). These results reveal that the electrocatalytic activity of UiO‐Pd‐E for the EOR is superior to that of the costly Pd/C (30 wt.%) catalyst. After the EOR reaction the catalyst was tested for the structural changes that might have evolved during the EOR. The XPS analysis was carried out and we did not find any significant change in the valence state of the Zr and Pd (Figure S10, Supporting Information), showing their structural robustness towards the EOR.

**Figure 8:**
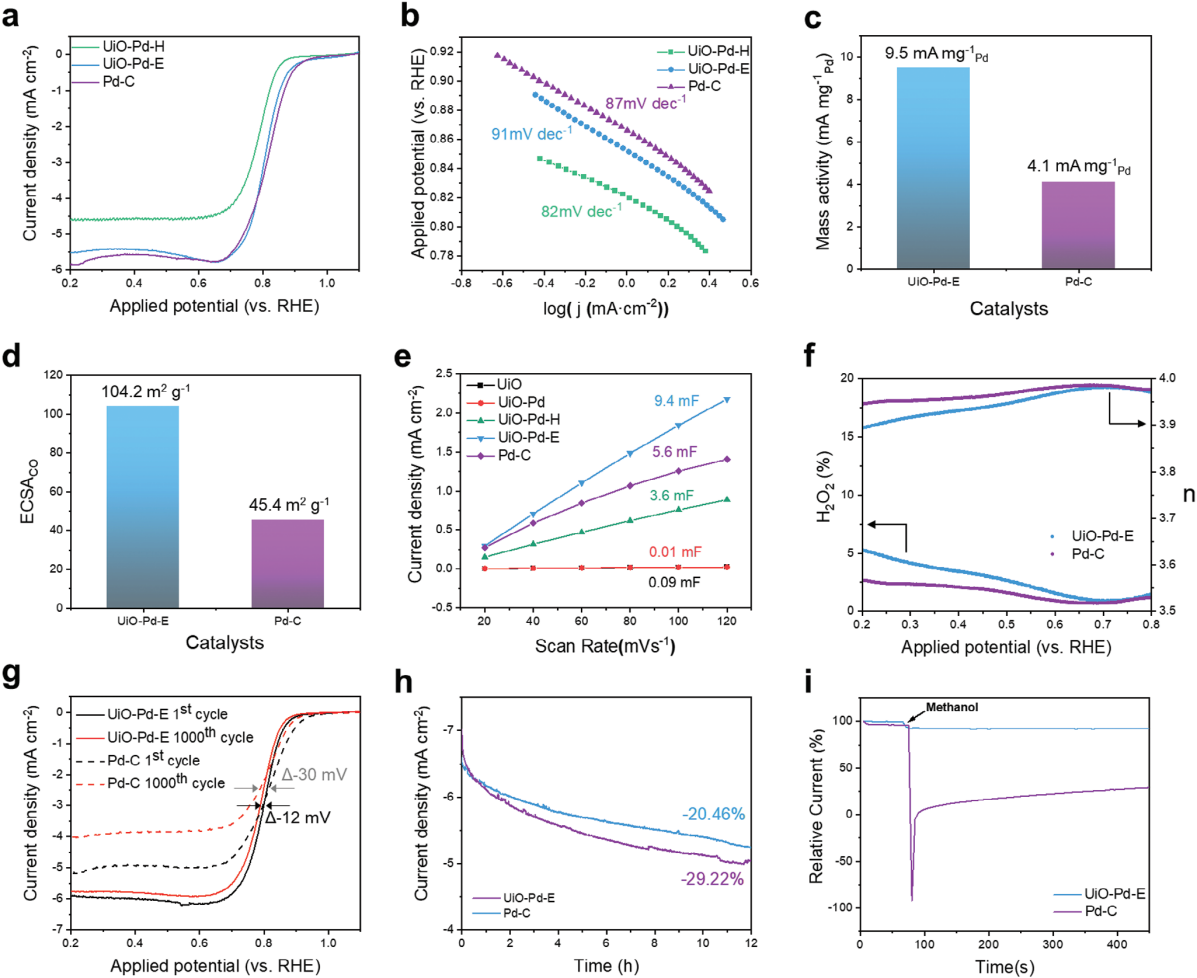
Oxygen reduction reaction. a) LSV curves of UiO‐Pd‐H, UiO‐Pd‐E, and Pd/C (30 wt.%) measured in an electrolyte containing 0.1 m KOH. b) Tafel plots of the samples obtained from the LSV curves. c) Comparison bar graphs for the Pd mass activity of UiO‐Pd‐E and Pd/C for ORR. d) Electrochemical active surface area (ECSA) of UiO‐Pd‐E and Pd/C obtained from CO‐saturated CV method. e) Electrical double layer capacitances (C_dl_) of the samples obtained from the capacitive CV curves. f) Rotating ring disk electrode (RRDE) testing results of the UiO‐Pd‐E and Pd/C. ORR durability test for UiO‐Pd‐E and Pd/C by g) continuous CV cycling and h) chronoamperometry methods. i) Methanol crossover testes by injecting 3.0 m methanol into the electrolyte.

Electrocatalytic oxidation and reduction reactions are crucial for energy‐conversion devices; thus, the electrocatalytic properties of UiO‐Pd‐E in the electrocatalytic oxygen reduction reaction (ORR) were also examined. **Figure** **8**a compares the ORR LSV polarization curves of UiO‐Pd‐E, UiO‐Pd‐H, and Pd/C (30 wt.%). Notably, the ORR activities of the UiO‐Pd‐E and Pd/C catalysts were similar, with both catalysts having an identical half‐wave potential (E_1/2_:0.81 V_RHE_) and similar limiting current densities (j_L_: 5.5 and 5.8 mA cm^−2^, respectively). In addition, the electrocatalytic ORR activity of UiO‐Pd‐E was superior to that of UiO‐Pd‐H (E_1/2_:0.78 V_RHE_, j_L_: 4.6 mA cm^−2^) (Figure 8a), owing to the in situ electrochemical evolution of Pd‐Zr alloys caged in the conductive carbon frames. The catalysts exhibited comparable Tafel slopes from 80 to 90 mV dec^−1^ (Figure 8b), indicating an identical rate‐determining step (RDS) in the electrocatalytic ORR. To calculate the Pd mass activity for the ORR, the current densities at 0.9 V_RHE_ were normalized to the Pd loading of the catalysts (Figure 8c), as determined by ICP‐OES. Importantly, the mass activity of UiO‐Pd‐E (9.5 mA mg^−1^_pd_) was more than double that of the commercial Pd/C catalyst (4.1 mA mg^−1^_pd_), demonstrating its promising practical potential and superior catalytic performance in the electrocatalytic ORR. A comparison of the catalytic activities of UiO‐Pd‐E in the EOR and ORR with those of recently reported electrocatalysts clearly demonstrates the outstanding performance of UiO‐Pd‐E (Tables S4 and S5, Supporting Information).

Furthermore, the electrochemically active surface areas (ECSA) of UiO‐Pd‐E (104.2 m^2^g^−1^) and Pd/C (45.4 m^2^g^−1^) were calculated using the hydrogen adsorption/desorption CV method (Figure 8d; Figure S11, Supporting Information). The increased ECSA of UiO‐Pd‐E indicates a higher number of active site. In addition, the electrical double‐layer capacitance (C_dl_) was also calculated via the CV method, which measures non‐Faradaic currents at different scan rates (Figure S12, Supporting Information)^[^ ^31^, ^32^ ^]^ C_dl_ is directly proportional to the ECSA of the catalyst. The C_dl_ of UiO‐Pd‐E (9.4 mF) was the highest among the samples (Figure 8e), and was ≈1.67 times higher than that of Pd/C (5.6 mF). This result is consistent with the ECSA determined from the CV method, confirming an increased number of active sites in UiO‐Pd‐E, likely owing to the intriguing nanocaged structure. In addition, the charge transfer number (*n*) of the ORR was measured using a rotating ring disk electrode (RRDE) to study the oxygen reduction pathways (Figure 8f).^[^ ^28^ ^]^ The UiO‐Pd‐E exhibited an *n* value of 3.9–4.0 in the examined potential window. Additionally, the H_2_O_2_ yield was lower than 5%, which is comparable to that of the commercial Pd/C catalyst. The *n* values obtained using the Koutecky‐Levich equation are consistent those obtained with the RRDE (Figure S13, Supporting Information). These results demonstrate that UiO‐Pd‐E follows a four‐electron pathway with excellent electrocatalytic ORR selectivity (2H_2_O + 4e^−^+ O_2_ → 4OH^−^).^[^ ^33^ ^]^

The electrocatalytic durability of UiO‐Pd‐E in the ORR was evaluated using various methods. Accelerated durability tests were performed in a saturated 0.1 m KOH electrolyte (Figure 8g). After 1000 continuous CV cycles in an electrocatalytic ORR potential window, UiO‐Pd‐E showed a slight shift in E_1/2_ (12 mV), which was substantially lower than that of the commercial Pd/C catalyst (ΔE_1/2_≅30 mV). Further, a chronoamperometry (*i*–t) test was performed at 0.6 V_RHE_ to further confirm the electrochemical stability of UiO‐Pd‐E. The initial current of UiO‐Pd‐E decreased by ≈20.5% after continuous ORR for 12 h (Figure 8h), whereas the Pd/C catalyst showed a higher activity loss indicated by a current reduction of 29.2%. The superior electrocatalytic durability of UiO‐Pd‐E in the ORR was ascribed to the Pd‐Zr nanoparticles caged in the carbon frames, which effectively prevented the aggregation of neighboring particles (Figure 3c). To further confirm the structural robustness after electrocatalytic ORR, we performed XPS analysis of UiO‐Pd‐E. In Zr 3d XPS spectra (Figure S14, Supporting Information) the peaks corresponds to the presence of Zr(0) is appeared ≈180 eV, whereas for Pd 3d also we observed the presence of Pd(0). This observation revealed that there is no structural change during the ORR, and the structure of the catalyst is robust.

ORR electrocatalysts must retain high electrocatalytic selectivity for cathodic reactions in a direct methanol fuel cell despite the oxidation of small molecules such as CH_3_OH. CH_3_OH can permeate across a polymer electrolyte membrane from the anode to the cathode, thereby significantly degrading the electrocatalytic activity owing to surface CO poisoning or undesired fuel oxidation reactions. The anti‐poisoning ability of UiO‐Pd‐E was evaluated by chronoamperometry at 0.6 V_RHE_ in an O_2_‐saturated alkaline electrolyte in the presence of CH_3_OH (Figure 8i). UiO‐Pd‐E exhibited excellent tolerance to CH_3_OH, as demonstrated by the negligible current drop, demonstrating exceptional electrocatalytic ORR selectivity and resistance to CO poisoning. In stark contrast, the initial current of the commercial Pd/C catalyst was almost completely removed, indicating poor CO poisoning resistance during the ORR. These results demonstrate the excellent catalytic activity, stability, and ORR selectivity of UiO‐Pd‐E even in poisoning environments. Thus, UiO‐Pd‐E successfully demonstrated multi‐functionality for diverse electrocatalytic reactions inducing the FAOR, EOR, and ORR, underscoring its broad applicability in diverse electrochemical devices.

## 3 Conclusion

UiO‐Pd‐E demonstrated exceptional electrocatalytic functionality in diverse electrochemical reactions, including the FAOR, EOR, and ORR, which are crucial in energy conversion devices. In particular, the Pd mass activity of UiO‐Pd‐E in the FAOR and ORR were 1.25 and two times greater, respectively, than those of the benchmark Pd/C (30 wt.%) catalyst. Furthermore, UiO‐Pd‐E exhibited excellent electrocatalytic durability in the electrocatalytic FAOR and ORR. The high performance of UiO‐Pd‐E in electrocatalytic reactions was attributed to the intriguing structure, in which Pd nanoparticles caged in the UiO carbon frames, and the simultaneous chemical alloying of Pd and Zr, which was achieved via in situ electrochemical reactions. The carbon frame cages in UiO‐Pd‐E effectively suppressed the aggregation of Pd nanoparticles during electrochemical reactions and thin interfacial carbon layers were newly formed. Simultaneously, the electronic structure of Pd was adjusted by Zr alloying elements, leading to a facile reaction pathway with superior charge transfer kinetics. Further, our electronic structure calculations propose a mechanism for the significantly enhanced direct FAOR on the Pd‐Zr catalyst. This improvement could be attributed to the surface charge dynamics of the Pd‐Zr surface and the distinct molecular interactions between Pd and Pd‐Zr surfaces, driven by the nucleophilic properties of O in HCOO and the electrophilic characteristics of C in COOH. This study offers a revolutionary strategy for the development of highly active and durable multifunctional electrocatalysts with high activity and durability in diverse chemical reactions.

## Conflict of Interest

The authors declare no conflict of interest.

## Supporting information



Supporting information

## Data Availability

The data that support the findings of this study are available from the corresponding author upon reasonable request.
