# Supplementary material for: Empowering Tri‐Functional Palladium's Catalytic Activity and Durability in Electrocatalytic Formic Acid Oxidation Reaction via Innovative Self‐Caging and Alloying Strategies
Source: Adv Sci (Weinh). 2024 Oct 14;11(46):2405725. doi: 10.1002/advs.202405725 (PMC11633530; doi:10.1002/advs.202405725)
Supplement: Supplementary file 1 — Supporting information [file ADVS-11-2405725-s001.docx]

*Supporting Information for*

**Empowering Tri-functional Palladium's Catalytic Activity and Durability in Electrocatalytic Formic Acid Oxidation Reaction via Innovative Self-Caging and Alloying Strategies**

*Chan-Woo Lee^1,†^, Sun Young Jung^2,†^, Jung Ho Ryu^3,†^, Gyeom Seong Jeon^1^, Ashish Gaur^4^, Min Su Cho^4^, Ghulam Ali^5^, Mingony Kim^6,7^, Kyung Yoon Chung^6,7^, Arpan Kumar Nayak^8^, Seoyoon Shin^9^,* *Jiseok Kwon^10^,* *Taeseup Song^10,*^, and Tae Ho Shin^9,*^, HyukSu Han^4,11,*^*

^1^Energy AI & Computational Science Laboratory, Korea Institute of Energy Research (KIER), Daejeon, 34129, Republic of Korea

^2^Department of Energy Engineering, Konkuk University, Seoul 05029, Republic of Korea

^3^Department of Materials Science and Engineering, Korea National University of Transportation, Chungju-si, Chungbuk, 27469, Republic of Korea

^4^Department of Energy Science, Sungkyunkwan University, Suwon 16419, Republic of Korea.

^5^U.S.-Pakistan Center for Advanced Studies in Energy (USPCASE), National University of Sciences and Technology (NUST), H-12, Islamabad, Pakistan

^6^Center for Energy Storage Research, Korea Institute of Science and Technology, Hwarang-ro 14-gil 5, Seongbuk-gu, Seoul 02792, Republic of Korea

^7^Division of Energy and Environment Technology, KIST School, Korea University of Science and Technology, Seoul 02792, Republic of Korea

^8^Regional Institute of Education, National Council of Educational Research and Training (NCERT), Mysore, 570006, India

^9^Korea Institute of Ceramic Engineering and Technology, KICET, 101 Soho-Ro, Jinju, 52851 Republic of Korea

^10^Department of Energy Engineering, Hanyang University, Seoul 04763, Republic of Korea.

^11^Department of Energy, Sungkyunkwan University, Suwon 16419, Republic of Korea.

*Corresponding author. Email: tssong@hanyang.ac.kr (T.S), ths@kicet.re.kr (T.H.S.), hyuksuhan@skku.edu (H.S.H),

†These authors contributed equally to this work.

**Materials and Methods**

**Chemicals**

Palladium (II) acetylacetonate (Pd(acac)_2_, 99%), 2,2′-bipyridine-5,5′-dicarboxylic acid (bpydc, 97%), N,N-Dimethylformamidecalcium (DMF, anhydrous, 99.8%), acetic acid (glacial, ReagentPlus®, ≥99%), hydrofluoric acid (HF, 48%) were purchased from Sigma-Aldrich (Germany). Zirconium(IV) chloride (ZrCl_4_, anhydrous, 98%) was purchased from Alfa Aesar (United States). Chloroform (99.5%) was purchased from Deajung Chemicals & Metals (Republic of Korea). All chemicals were used as purchased without further treatments.

**Materials Synthesis**

**Synthesis of UiO**

ZrCl_4_ (0.23304g) and 2,2′-bipyridine-5,5′-dicarboxylic acid (bpydc, 0.06105g) were dissolved in a mixed solution of acetic acid (5 mL) and DMF (20 mL). The solution was stirred about for 8h to completely dissolve the powders. Then, the mixed solution was transferred into a Teflon-lined autoclave, which was hydrothermally reacted at 100ºC for 24h. The product was collected and washed with chloroform and DMF several times. The resulting powders were again dispersed in chloroform solution and stirred for 6 h. Finally, the sample was washed with chloroform several times and dried in an oven at 80°C overnight to obtain the UiO.

**Synthesis of UiO-Pd**

The synthesis of UiO-Pd was identical for the preparation of UiO, except that Pd(acac)_2_ (0.0366g) was added to the mixed solution as a Pd precursor.

**Synthesis of UiO-Pd-H**

The prepared UiO-Pd powder was placed in an alumina crucible, which was placed in a center of the Ar-purged tube. The tube furnace was heated from room temperature to temperature of 850°C using a heating rate of 2°C/min under Ar and then dwelled for 2 h. Subsequently, the furnace was cooled down to room temperature to obtain the UiO-Pd-H.

**Synthesis of UiO-Pd-E**

UiO-Pd-H was soaked in a 5% HF solution at 60°C with a continuous stirring for about 1h. Then, the collected powder was washed with ethanol and DI water several times. Subsequently, the washed powder was dried in an oven at 80 °C to obtain the UiO-Pd-E.

**Characterizations**

**Scanning electron microscopy** **(SEM)**

FE-SEM (model S4800; Hitachi) was performed on the samples for examination of morphologies and structural parameters.

**Transmission electron microscopy** **(TEM)**

TEM (Talos F200X; Thermo Fisher Scientific) was used to obtain high-resolution TEM images with sub-nano scale. In addition, elemental distributions on the samples were imaged using scanning-TEM (STEM) energy dispersive X-ray (EDX) spectroscopy.

**Powder X-ray diffraction (XRD)**

XRD was performed using a X-ray diffractometer (D/Max-2500/PC, Rigaku) at 40 kV and 100 mA. A Cu-Kα radiation (λ = 0.15418 nm) source was used. Crystal structures of the samples were analyzed from the collected XRD data.

**X-ray photoelectron spectroscopy** **(XPS)**

Surface electronic configurations of the samples were studied by XPS (VG ESCALAB 200i, Thermo Fisher Scientific, United States). Energy calibration was conducted using a C 1s peak position as a reference. Survey and high resolution XPS scans were processed with pass energies of 100 and 20 eV respectively.

**Inductively coupled plasma-optical emission spectroscopy (ICP-OES)**

The amount of Pd in the samples was calculated by means of ICP-OES (Thermo Scientific; iCAP6500 Duo). The RF power and wavelength were set at 1350 W and 214.423 nm, respectively. Samples were first dissolved in a nitric/hydrochloric acid mixture and heated at 150°C for approximately 30 min. Subsequently, hydrofluoric acid was added to complete dissolution of all materials and heated at 150 °C until the solution became transparent.

**Fourier-transform infrared (FTIR)**

FTIR spectroscopy (FT/IR-4100; Jasco) was performed to investigate the nature of the chemical bonds in the samples.

**Thermogravimetric analysis (TGA)**

TGA (SDT Q600, TA instruments, United States) was performed to measure the mass change of the samples as temperature varies. The temperature was increased using a heating rate of 10°C/min up to 800°C under nitrogen.

**Brunauer emmett teller analysis (BET)**

Specific surface area and pore size of the samples were obtained through BET (BELSORP-MAXG, Microtrac BEL, Japan) analysis. The BET surface area was measured using the amount of N_2_ gas adsorbed on the surface of the sample.

**Electrochemical Measurements**

**Preparation of catalyst inks and electrodes**

Catalyst ink was prepared by dispersing 5 mg catalyst powers in a mixed solution of 750 µL DIW, 250 µL ethanol, 20 µL Nafion 117 solution. After 20 min sonication, a 5 µL of catalyst ink was applied to a clean surface of glassy carbon electrode (GCE, 3 mm diameter), giving a catalyst loading amount of approximately 0.35 mg cm^-2^. The GCE was then dried under air at room temperature.

**Evaluation of electrocatalytic FAOR performance**

The electrocatalytic FAOR properties of the samples were measured in an electrolyte of 0.1 M CH₂O₂ + 0.1 M HClO_4_ using a three-electrode cell connected to a potentiostat (model Autolab PGSTAT; Metrohm). A rotating disk electrode (RDE) was used for the investigation of the electrochemical properties of the catalysts. A typical three-electrode set-up (Pt as the counter electrode and Ag/AgCl as the reference electrode) was used for the electrochemical tests. The recorded potentials were then recalculated in relation to the reversible hydrogen electrode (RHE). All of the electrocatalysts were first subjected to cyclic voltammetry (CV) scans at a scan rate of 50 mV s^-1^ in Ar saturated 0.1 M HClO_4_. FAOR activity was then assessed by a CV scan using a scan rate of 10 mV s^-1^ with a rotation speed of 1600 rpm. In addition, electrochemical impedance spectroscopy (EIS) was performed at 0.6 V_RHE_. For testing durability of catalysts, a chronoamperometry test was performed at 0.3 V_RHE_.

**CO-stripping measurement**

The working electrode was first immersed in a 0.1 M HClO_4_ solution saturated with CO at a holding potential of 0.45 V for 1h to ensure complete adsorption of a monolayer of CO molecules. A CO stripping voltammogram (CSV) was obtained by immediately transferring the electrode into a 0.1 M HClO_4_ solution saturated with N_2_ gas. Subsequently, the measurement was performed by varying the voltage between 0 and 1.1 V at a rate of 50 mVs^-1^.

**Evaluation of electrocatalytic EOR performance**

A three-electrode cell connected to a potentiostation (model Autolab PGSTAT; Metrohm) was used to measure the electrocatalytic EOR properties of the samples in 1.0 M KOH and 1.0 M C_2_H_5_OH electrolyte. The electrolyte was purged with Ar gas for approximately 20 min. All of the electrocatalysts were first subjected to CV scans for 50 cycles at a scan rate of 50 mV s^-1^. Linear sweep voltammetry (LSV) curves were then recorded at a scan rate of 10 mV s^-1^ using a rotation speed of 1600 rpm.

**Evaluation of electrocatalytic ORR performance**

A three-electrode cell connected to a potentiostation (model Autolab PGSTAT; Metrohm) was used to measure the electrocatalytic ORR properties of the samples in a 0.1M KOH electrolyte. The electrolyte (0.1 M KOH) was purged with O_2_ gas for approximately 20 min. All electrochemical measurements were conducted under O_2_ gas purging conditions. In addition, 100 cycles of CV scans were performed at a scan rate of 100 mV s^-1^ in the ORR potential window to electrochemically stabilize the catalyst surface. LSV curves were then recorded at a scan rate of 5 mV s^-1^ using different rotating speeds. Tafel plots were derived from the LSV polarized curves, where the Tafel slopes can be calculated using the equation: η = b log j + a where (b: Tafel slope, j: current density, η: overpotential).

**Determination of electrochemical surface area**

Electrochemical surface area (ECSA) of the samples was determined by using the following equation,

ECSA = Q / (0.21 × m_Pd_) (1)

,where Q (mC) is the charge for the adsorption/desorption of hydrogen, which can be obtained from CV curves. Specifically, the integrated area for hydrogen adsorption peaks was divided by the scan rate (0.01 V s^-1^ in our work), which is corresponding to the value of Q. m_Pd_ is the mass of Pd in the catalyst loaded on the electrode, which can be obtained from the ICP-OES results. 0.21 (mC cm^-2^) is the electric charge associated with the adsorption of hydrogen on the surface of Pd in a monolayer.

**Determination of double layer capacitance**

CV scans were conducted within a non-Faradic potential range (e.g., 1.02 ~ 1.14 V_RHE_) at various scan rates (20 - 120 mV/s) in a 0.1M KOH electrolyte to determine the double-layer capacitance (C_dl_) of the catalysts. The difference between anodic and cathodic currents (ΔJ = J_anodic_ - J_cathodic_) at the midpoint potential (1.08 V_RHE_) was plotted against the scan rate. The slope of the resulting plot corresponds to twice the C_dl_ of the catalyst. The equation utilized for calculating C_dl_ is as follows,

*C_dl_* = $\frac{j_{a{- j}_{c}}}{2 \times v}$ = $\frac{j_{a}+\left| j_{c} \right|}{2 \times v}$ = $\frac{\Delta j}{2 \times v}$ (2)

, where, *j_a_* and *j_c_* represent the anodic and cathodic current densities, respectively, recorded at the midpoint of potential, 𝜐 is a scan rate.

**Measurement of H_2_O_2_ production rate and electron transfer number using rotating ring disk electrode (RRDE)**

The electrolyte (0.1 M KOH) was purged with O_2_ gas for about 30 min. The measurement was started after turning off all gas supply. The LSV was conducted using a scan rate of 5 mV/s with a rotation speed of 800 rpm while collecting disk and ring currents in a RRDE mode. The H_2_O_2_ production rate and electron transfer number can be determined as,

$H_{2}O_{2} \left( \varepsilon\right)\%=\frac{Number of O_{2} involved in H_{2}O_{2} generation}{Number of O_{2} supplied}=\frac{2I_{H_{2}O_{2}}}{I_{H_{2}O}+2I_{H_{2}O_{2}}}=\frac{2\frac{I_{r}}{n}}{I_{d}+\frac{I_{r}}{n}}\times100$ (3)

$electron transfer number (n)=4-\frac{\varepsilon}{100}\times2$ (4)

, where I_r_ and I_d_ are ring and disk currents, respectively, and have the below relations.

$I_{d}=I_{H_{2}O}+I_{H_{2}O_{2}}$ (5)

$I_{r}=n\cdot I_{H_{2}O_{2}}$ (6)

**Electrochemical stability and methanol crossover for ORR**

Chronoamperometry was performed at 0.6 V_RHE_ using a rotating speed of 1600 rpm. For testing methanol crossover, 1 ml of 3 M methanol was injected during chronoamperometry measurement.

**Electronic Structure Calculations of HCOO and COOH adsorbates on Pd and Pd-Zr surfaces**

Density functional theory (DFT) calculations were performed utilizing the Vienna Ab initio Simulation Package (VASP), employing the projector-augmented wave (PAW) method in conjunction with the Perdew-Burke-Ernzerhof (PBE) functional [D1-3]. A convergence test for plane-wave cutoff energy and K-points (Monkhorst-Pack grids) was conducted with a 0.01 eV/atom criterion. Electronic and ionic relaxations were performed based on criteria of 10^-6^ eV and 0.02 eV/Å, respectively [D4].

Based on the bulk structure of palladium (Pd), an asymmetric surface slab model of Pd (111) was constructed with a thickness of five atomic layers (Fig. S10a). All Pd atom positions were fully relaxed within fixed supercell, except for the bottom two layers, which were held fixed. A Pd-Zr (111) surface slab was constructed by replacing four out of twelve top-layer Pd atoms with Zr atoms, where the Zr atoms were evenly distributed among the Pd atoms, achieving a Pd/Zr ratio of 2 (Fig. S10b).

To compare direct and indirect FAOR, adsorption energetics of HCOO* (direct) and COOH* (indirect) have been predicted based on following equation:

Ead (M) = E(Surface + M*) - E(surface) – E(M), (7)

where *E*_ad_ (M) is adsorption energy of the M (HCOO, COOH) on the surface (Pd surface, Pd-Zr surface). If the *E*_ad_ (M) is exothermic (negative), low energy indicates high stability of adsorbed M (M*). Stable molecular geometries of the HCOO* and COOH* were determined by comparing multiple configurations of the molecules on Pd and Pd-Zr surface models.

**References**

D1. Kresse, G.; Furthmüller, J., Efficient iterative schemes for ab initio total-energy calculations using a plane-wave basis set. Physical review B 1996, 54 (16), 11169.

D2. G. Kresse and D. Joubert, From ultrasoft pseudopotentials to the projector augmented-wave methodPhys. Rev. B 59, 1758 (1999).

D3. Perdew, J. P.; Burke, K.; Ernzerhof, M., Generalized gradient approximation made simple. Physical Review Letters 1996, 77 (18), 3865.

D4. H. J. Monkhorst and J. D. Pack, Phys. Rev. B 13, 5188 (1976). Special points for Brillouin-zone integrations


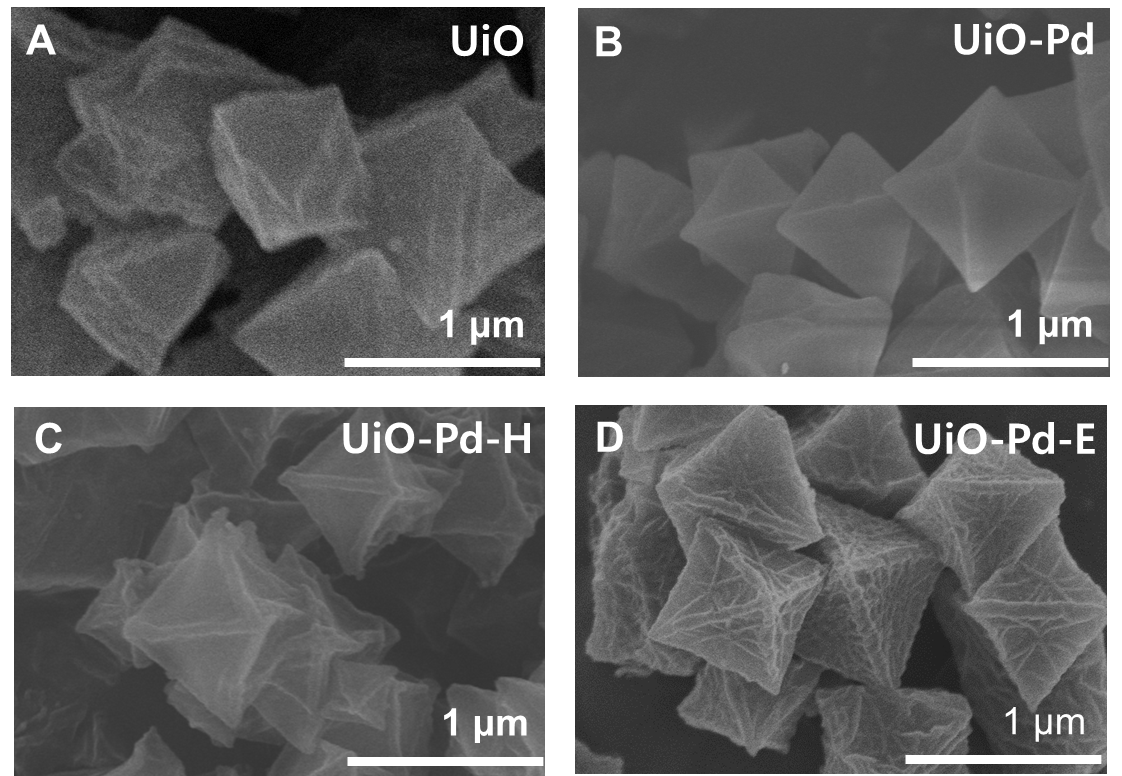


**Figure S1**. SEM images of (a) UiO, (b) UiO-Pd, (c) UiO-Pd-H, and (d) UiO-Pd-E, respectively.


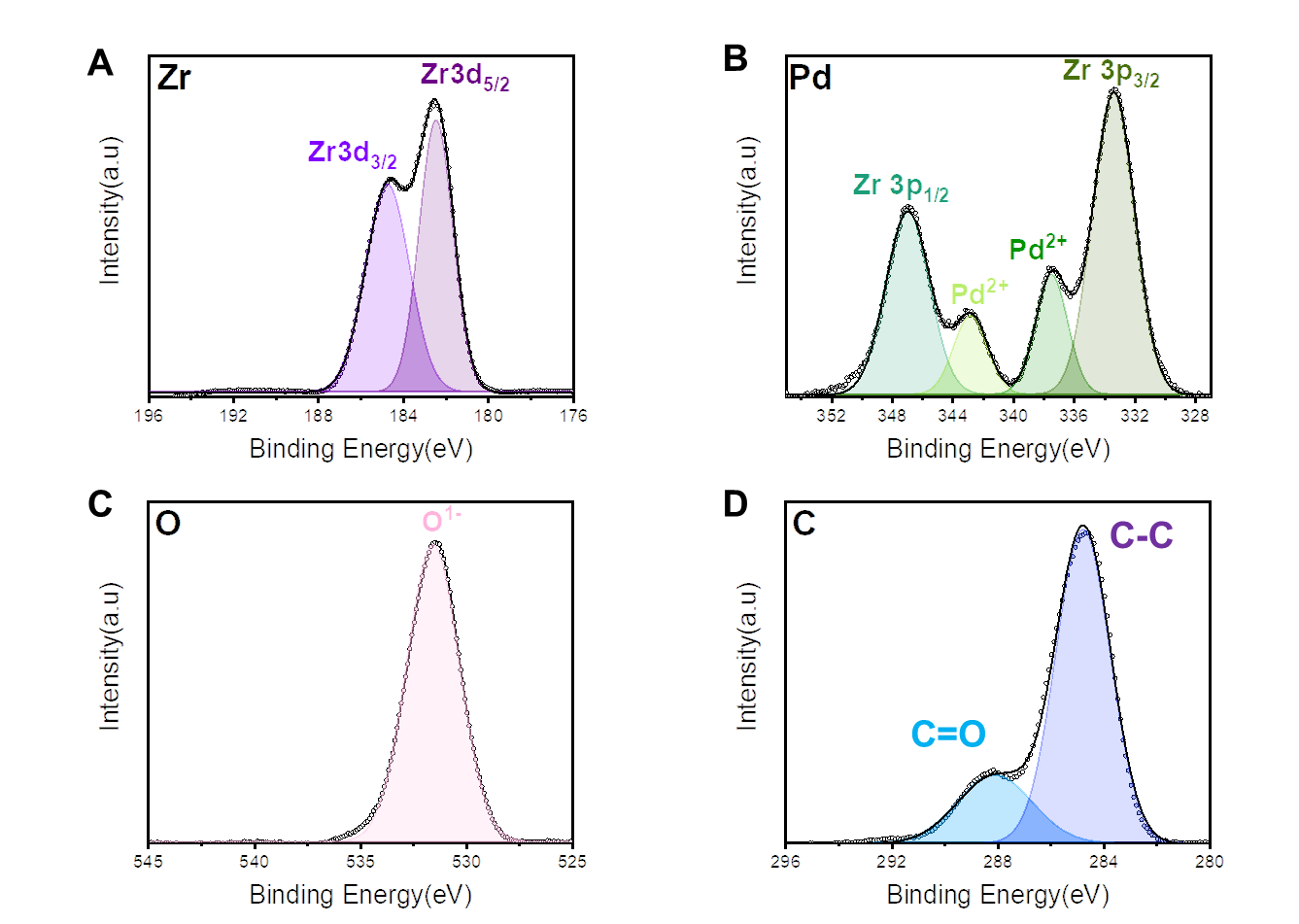


**Figure S2.** XPS spectra of (a) Zr, (b) Pd, (c) O and (d) C in UiO-Pd.





**Figure S3.** N1s XPS spectra present in the UiO-Pd-E


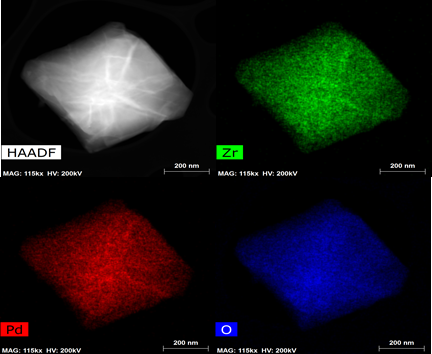


**Figure S4.** STEM-EDX mapping images of UiO-Pd.


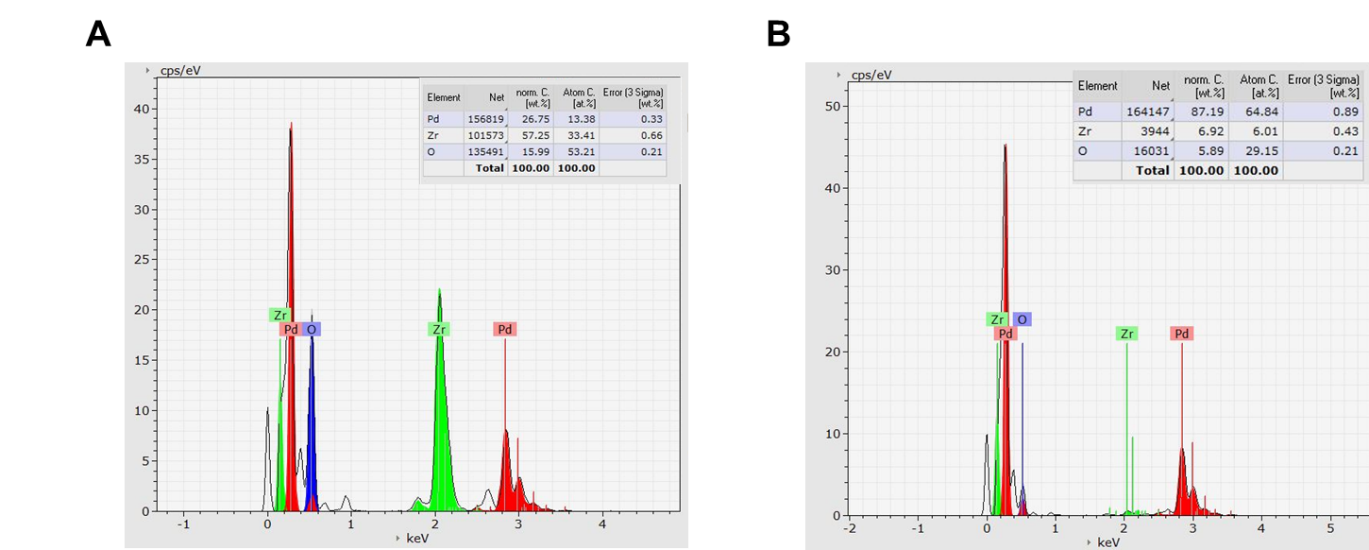


**Figure S5.** Quantitative STEM-EDX mapping results for (a) UiO-Pd-H and (b) UiO-Pd-E.


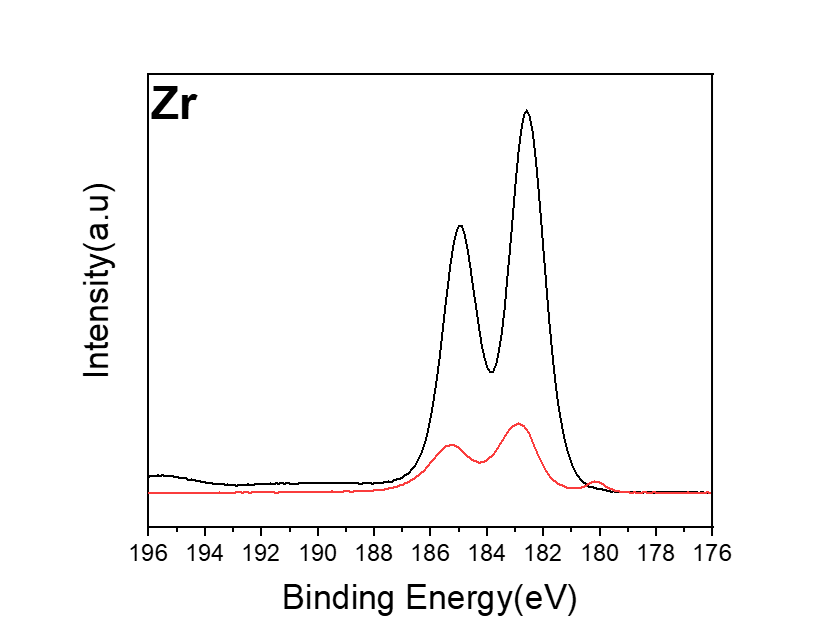


**Figure S6.** XPS spectra of Zr 3d in UiO-Pd-H (black) and UiO-Pd-E (red).


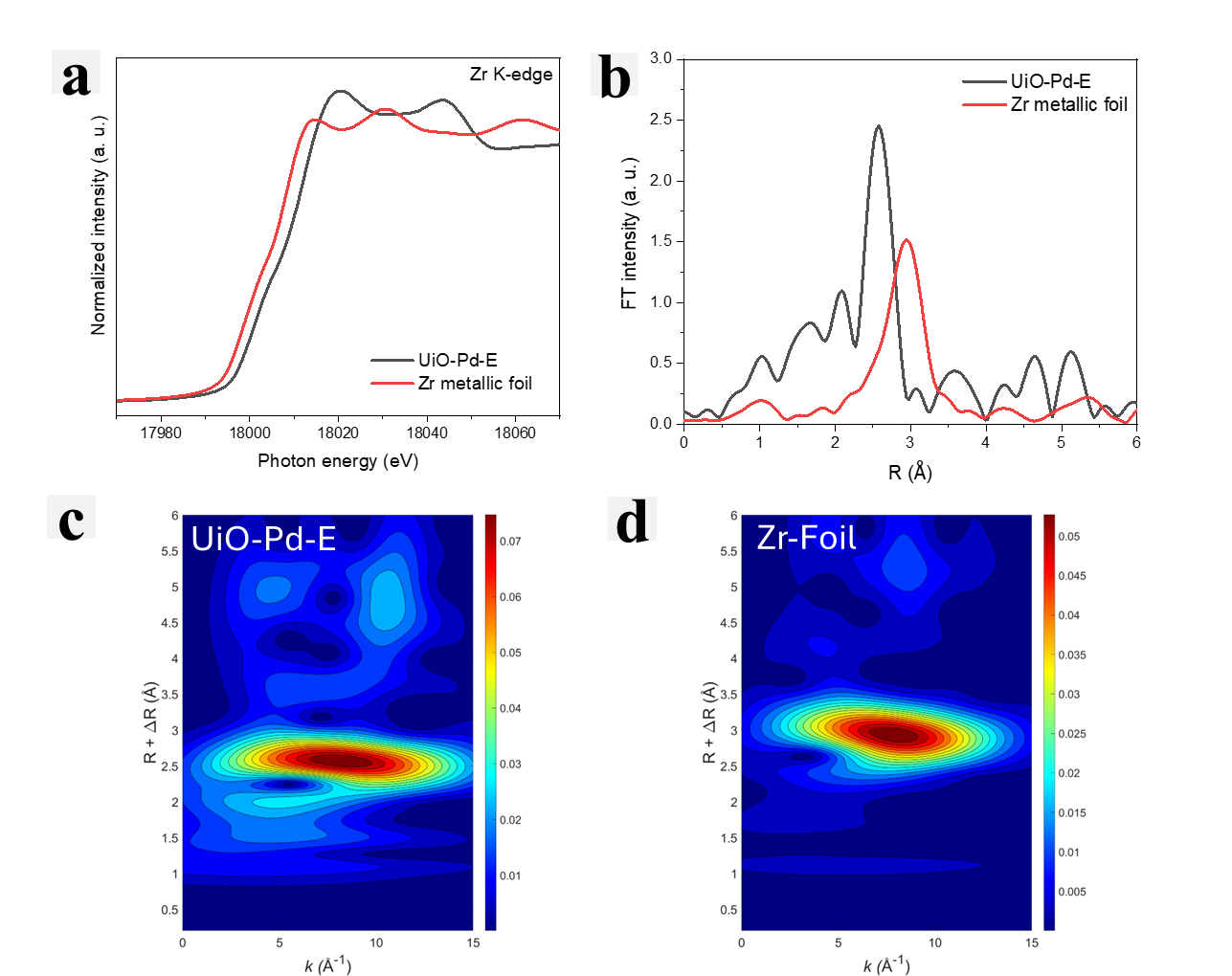


**Figure S7.** (a) X-ray absorption near edge spectra (XANES) of Zr-Kedge , (b) EXAFS spectra of Zr-K edge (c) CCWT plot of UiO-Pd-E and (d) Zr-foil


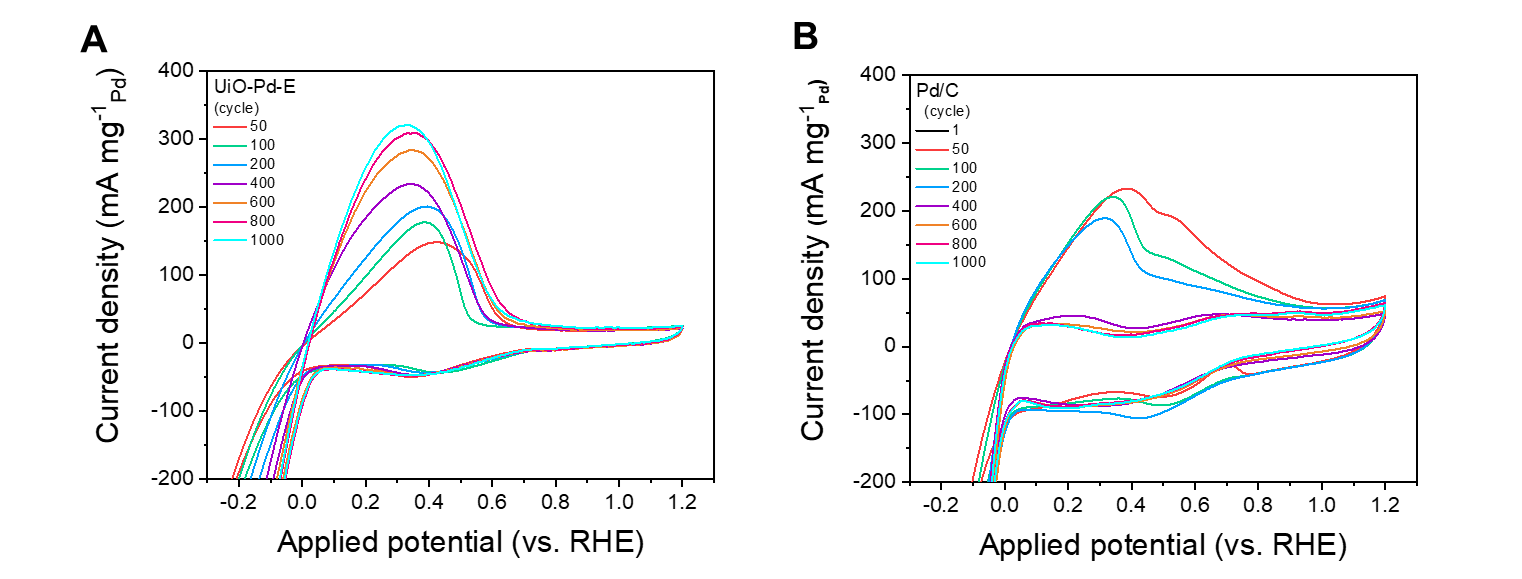


**Figure S8.** Cycling FAOR performances of (a) UiO-Pd-E and (b) Pd/C measured in an electrolyte containing 0.1 M CH₂O₂ and 0.1 M HClO_4_ at a scanning rate of 50 mV s^–1^.

**Figure S9.** (a) Asymmetric surface slab model of Pd (111) constructed with a thickness of five atomic layers. (b) Asymmetric surface slab model of Pd-Zr (111) constructed by replacing four out of twelve top-layer Pd atoms with Zr atoms (Pd/Zr ratio of 3), where the Zr atoms are evenly distributed among the Pd atoms.


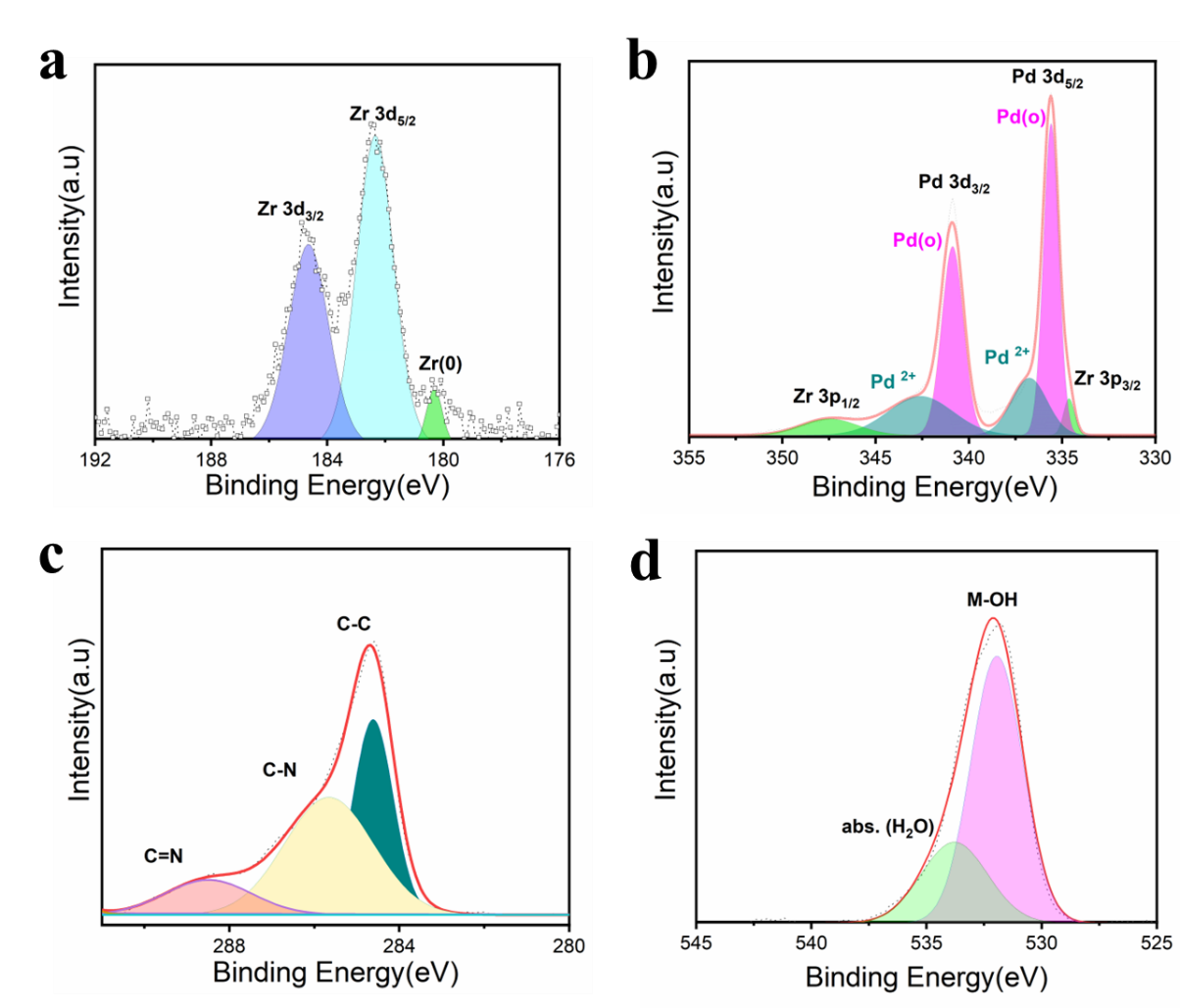


**Figure S10.** XPS spectra of (a) Zr3d, (b) Pd 3d, (c) C1s and (d) O1s present in UiO-Pd-E after EOR


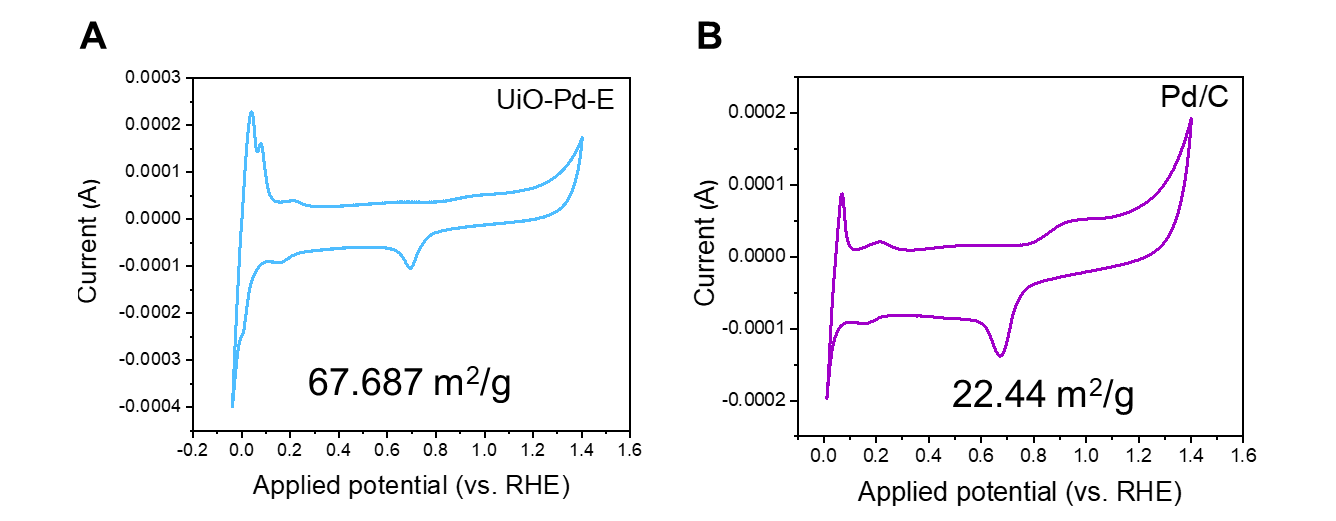


**Figure S11.** CV scans for UiO-Pd-E and Pd/C in N_2_-saturated 0.1 M HClO_4_ solution at a scan rate of 10 mV s^−1^.


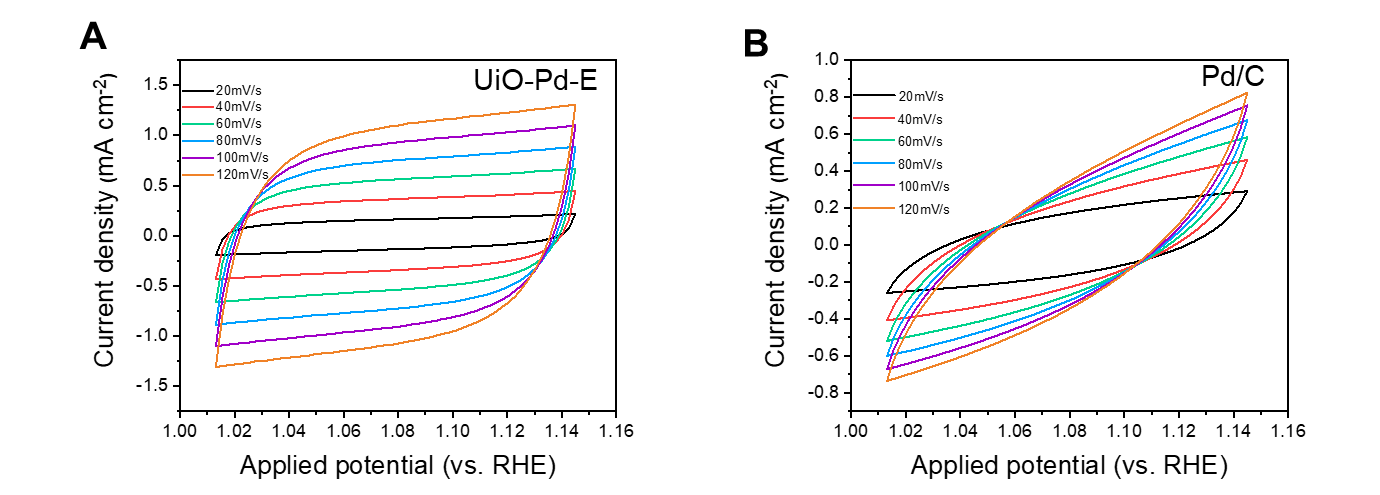


**Figure S12.** CV scans of (a) UiO-Pd-E and (b) Pd/C measured at different scan rates in an electrolyte containing 0.1M KOH.


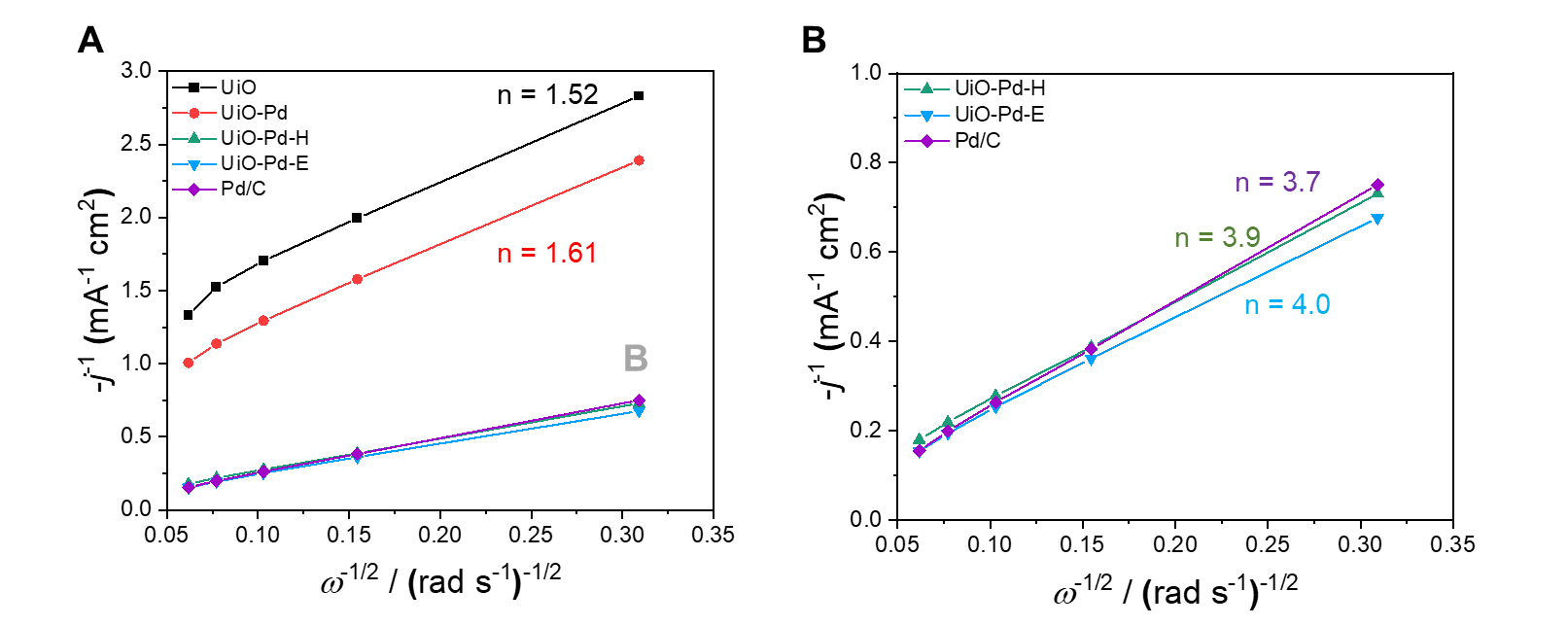


**Figure S13.** (a) K-L plots UiO, UiO-Pd, UiO-Pd-H, UiO-Pd-E, and Pd/C measured in an electrolyte containing 0.1M KOH, and (b) the calculated electron transfer numbers.


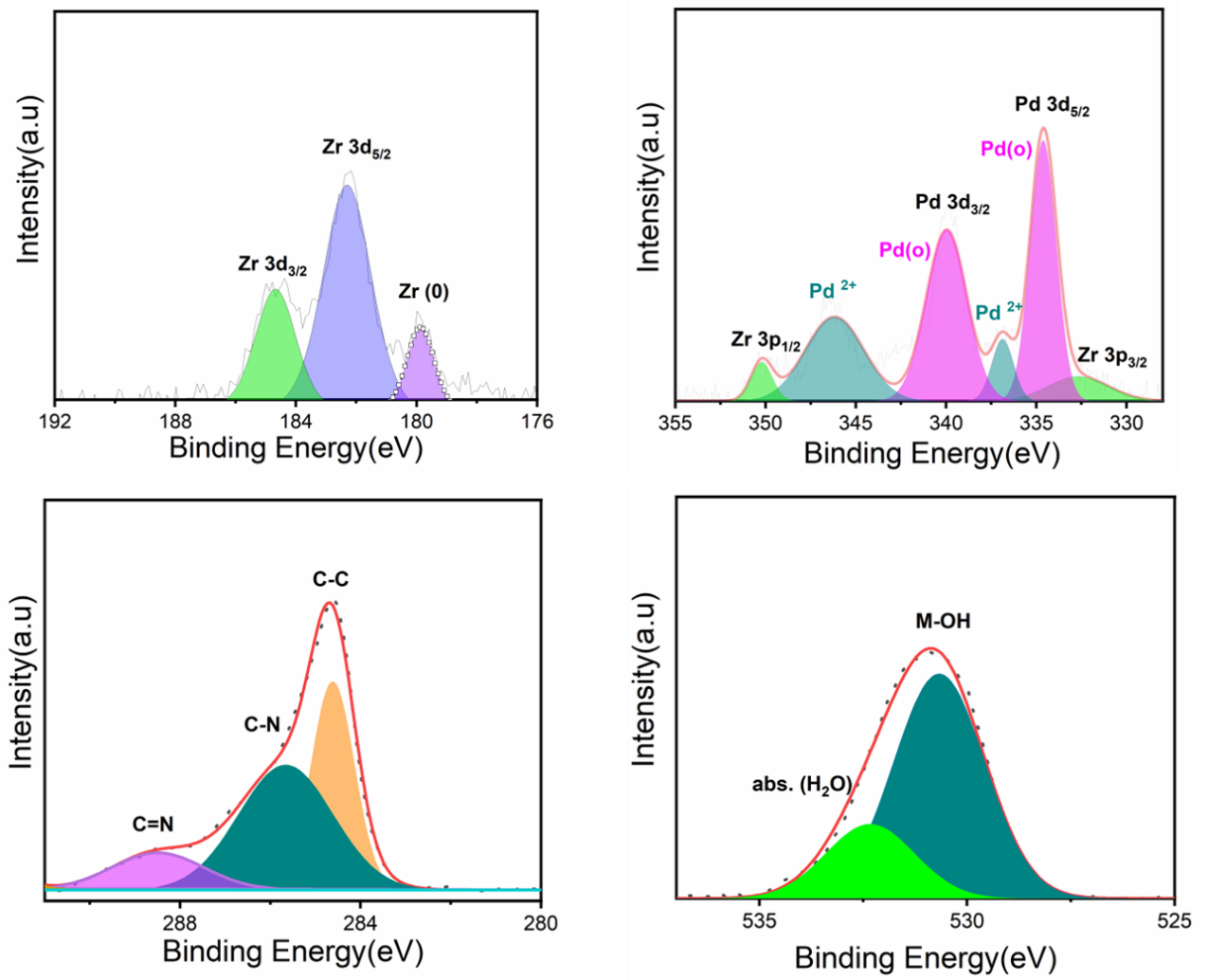


**Figure S14**. XPS spectra of (a) Zr 3d, (b) Pd 3d, (c) C1s and (d) O1s present in UiO-Pd-E after the electrocatalytic ORR stability test.

|  | Pd(wt%) | Zr(wt%) |
| --- | --- | --- |
| UiO-Pd-H | 12.9 | 31.6 |
| UiO-Pd-E | 24.2 | 6.8 |

**Table S1.** ICP-OES data of the UiO-Pd-H and UiO-Pd-E

|  | Pd (㎎/㎏) | Zr(㎎/㎏) |
| --- | --- | --- |
| **Before FAOR**  **1000cycle** | **< 0.5** | **< 0.5** |
| **After FAOR**  **1000cycle** | **< 0.5** | **< 0.5** |

**Table S2:** ICP-OES data of UiO-Pd-E before and after the FAOR

**Table S3.** A **c**omparison of FAOR catalytic performance.

| **Catalyst** | **Electrolyte** | **Scan rate**  **(mV/s)** | **Peak potential** | **Peak current density**  **(mA/cm^2^)** | **Peak Mass activity**  **(A/mg _metal_^-1^)** | **Loading amount**  **(mg/cm^2^)** | **Reference** |
| --- | --- | --- | --- | --- | --- | --- | --- |
| UiO-Pd-E | **0.1M HClO4**  **+ 0.1M HCOOH** | 10 | 0.36V  vs. RHE | 19 | 0.264 A/mg_Pd_ | 0.35 | This work |
| Intermetallic  (Ir_0.9_Pd_0.1_)_3_V/C | **0.5M H2SO4**  **+ 0.5M HCOOH** | 20 | 0.75 V  vs. RHE | - | ~0.25 A/mg_(Ir+Pd)_ | - | ^[1]^ |
| PtCo  Tetrahexahedral nanoparticles | **0.5M H2SO4**  **+ 0.5M HCOOH** | 50 | 0.62 V  vs. RHE | 17.5 | 0.21A/mg_Pd_ | 2.5 | ^[2]^ |
| Pd-nanoarray@CP | **0.1M H2SO4**  **+ 0.1M HCOOH** | 50 | 0.54 V  vs. RHE | - | 0.262 A/mg_Pd_ | - | ^[3]^ |
| Nanoporous Pd | **0.5M H2SO4**  **+ 0.5M HCOOH** | 10 | - | - | 0.2 A/mg_Pd_ at 0.27 V | - | ^[4]^ |
| Pd/CNT | **0.5M H2SO4**  **+ 0.5M HCOOH** | 50 | 0.44 V  vs. RHE | - | 0.17 A/mg_Pd_ | - | ^[5]^ |
| PdSn/C | **0.5M H2SO4**  **+ 1M HCOOH** | 10 | - | - | 0.057 A/mg_Pd_ | - | ^[6]^ |
| Pd black | **0.5M H2SO4**  **+ 0.5M HCOOH** | 50 | - | - | - | - | ^[7]^ |
| Pd–Au/C | **0.5M H2SO4**  **+ 0.5M HCOOH** | - | 0.37 V  vs. RHE | 18.6 | 0.25 A/mg_Pd_ | - | ^[8]^ |
| Pd_7_Ru_3_/C | **0.5 M HClO4**  **+ 0.5 M HCOOH** | 50 | - | - | 0.2 A/mg_Pd_ | - | ^[9]^ |
| 1-PtPd/CNTs | **0.5M H2SO4**  **+ 0.5M HCOOH** | 50 | 0.35 V  vs. RHE | - | 0.22 A/mg_Pd_ | - | ^[10]^ |
| Pd nanowires/C | **0.5M HClO4**  **+ 0.5M HCOOH** | 50 | 0.4 V  vs. RHE | 13.89 | 0.25 A/mg_Pd_ | - | ^[11]^ |
| 3DOM Pd | **0.5M H2SO4**  **+ 0.5M HCOOH** | 50 | 0.23 V  vs. RHE | - | 0.13 A/mg_Pd_ | - | ^[7]^ |
| Flower-like Pd nanodendrites | **0.1M HClO4**  **+ 0.1M HCOOH** | 50 | 0.08 V  vs. RHE | - | 0.187 A/mg_Pd_ | - | ^[12]^ |
| Pd DNs | **0.5M H2SO4**  **+ 0.5M HCOOH** | - | 0.21 V  vs. RHE | - | - | - | ^[13]^ |
| Pd-Cu/C | **0.5M H2SO4**  **+ 0.5M HCOOH** | 20 | 0.4 V  vs. RHE | 3.2 | - | 0.127 | ^[14]^ |
| Pd/GF with 25 vol.% Triton X-102 | **0.5M H2SO4**  **+ 1M HCOOH** | 5 | - | 8 | - | 0.4 | ^[15]^ |

**Table S4.** A **c**omparison of EOR catalytic performance.

| **Catalyst** | **Electrolyte** | **Peak current**  **Density**  **(mA/cm^2^)** | **Onset potential** | **Peak potential** | **Peak Mass activity**  **(A/mg _metal_^-1^)** | **Loading amount**  **(mg/cm^2^)** | **Reference** |
| --- | --- | --- | --- | --- | --- | --- | --- |
| UiO-Pd-E | 1M KOH  + 1M EtOH | 60.32 | 0.579 V | 0.850 | 0.71 A/mg_Pd_ | 0.35 | This work |
| Pt-Cu Nanosheet Alloy | 0.5M H_2_SO_4_  + 0.1M EtOH | - | - | - | 0.7 A/mg_Pt_ | - | ^[16]^ |
| Pt_90_Sm_10_ alloy NCs | 0.1M HClO_4_  + 0.1M EtOH | - | - | - | 0.17 A/mg_Pt_ | - | ^[17]^ |
| Pt0.25Mn_0.75_-2X-700°C-1 h | 0.5M H_2_SO_4_  + 0.1M EtOH | 8.4 | 0.24 V | 0.71 |  | 0.11 | ^[18]^ |
| ZrO2/NiO/rGO | 0.5M KOH  + 0.5M EtOH | 17.3 | - | 0.52 |  | - | ^[19]^ |
| Ni_3_S_4_–NiS-rGO | 1M KOH  + 0.5M EtOH | 11 | - | 0.59 |  | - | ^[20]^ |
| Pd_3_Pb_P10s | 0.5M KOH  + 1M EtOH | - | - | - | 0.51 A/mg_Pd_ | - | ^[21]^ |
| Cu_1_Pd_2_/C | 0.5M KOH  + 0.5M EtOH | - | - | -0.3 | 0.52 A/mg_Pd_ | - | ^[22]^ |
| Pd_3_Cu | 1M KOH  + 1M EtOH | - | 0.46 V | 0.75 | 0.35 A/mg_Pd_ | 0.2 | ^[23]^ |
| PtPd NPs | 0.5M H_2_SO_4_  + 0.5M EtOH | - | - | - | 0.31 A/mg_metal_ | - | ^[24]^ |
| PdAg/CNT | 1M KOH  + 0.1M EtOH | - | - | - | 0.305 A/mg_metal_ | - | ^[25]^ |

**Table S5.** A **c**omparison of ORR catalytic performance.

| **Catalyst** | **Electrolyte** | **Mass activity**  **(0.85V_RHE_)** | **Tafel slope (mV/dec)** | **Onset potential (V_RHE_)** | **Half wave potential (V_RHE_)** | **Electron transfer number (N/A)** | **Reference** |
| --- | --- | --- | --- | --- | --- | --- | --- |
| UiO-Pd-E | 0.1M KOH | 33.8 A/g_Pd_ | 91 | 0.91 | 0.81 | 3.89~3.97 | This work |
| Pt NAs | 0.5 M H_2_SO_4_ | 12.4 A/g_Pt_ | - | - | 0.839 | - | ^[26]^ |
| Pd@Pt core shell nanostructures | 0.5 M H_2_SO_4_ | 14.8 A/g_Pd_ | - | - | 0.862 | - | ^[27]^ |
| Pt–Ni/graphene | 0.5 M H_2_SO_4_ | 0.14 A/ g_Pt_  (0.8V_RHE_) | - | 0.98 | 0.77 | 3.95 | ^[28]^ |
| Pt/GO-C | 0.5 M H_2_SO_4_ | 0.005 A/g_Pt_ | 64 | - | 0.79 | 3.9 | ^[29]^ |
| PICL | 1M HClO_4_ | 2.17 A/g_Pd_ | - | 0.94 | - | - | ^[30]^ |
| 35nm AgNW | 0.1M KOH | 7.2 A/g | 100 | - | 0.649 | - | ^[31]^ |
| RGO-ZnCo_2_O_4_ | 1M KOH (1200rpm) | 29.2 A/g | - | 0.95 | 0.87 | 3.7-3.95  (K-L plot) | ^[32]^ |
| PdCub_3_-20 | 0.5 M H_2_SO_4_  (1900rpm) | 17 A/g_Pd_ | 119 | - | 0.67 | - | ^[33]^ |
| Ag-Pt-Ag | 0.1 M NaOH | 1.08 A/g_Pt_ | 140 | - | - | - | ^[34]^ |
| Pt@NiXL | 1M KOH | 29 A/g_Pt_ | 129 | - | - | - | ^[35]^ |
| NR-Co_2_P TOPO | 0.1M KOH | 28.3 A/g | - | 0.83 (0.9V_RHE_) | 0.78 | 3.98-4.18  (K-L plot) | ^[36]^ |

**References in SI**

[1] T. Shen, S. Chen, C. Zhang, Y. Hu, E. Ma, Y. Yang, J. Hu, D. Wang, *Advanced Functional Materials* **2022**, 32, 2107672.

[2] L. Huang, C. Y. Zheng, B. Shen, C. A. Mirkin, *Advanced Materials* **2020**, 32, 2002849.

[3] Y. Zhou, Y. Yang, X. Zhu, T. Zhang, D. d. Ye, R. Chen, Q. Liao, *Advanced Functional Materials* **2022**, 32, 2201872.

[4] X. Wang, W. Wang, Z. Qi, C. Zhao, H. Ji, Z. Zhang, *Electrochemistry Communications* **2009**, 11, 1896.

[5] O. Winjobi, Z. Zhang, C. Liang, W. Li, *Electrochimica Acta* **2010**, 55, 4217.

[6] B. Wu, B. Wang, C. Deng, Y. Gao, *Applied Catalysis B: Environmental* **2011**, 103, 163.

[7] S. Tsang, *Chemical Communications* **2011**, 47, 7389.

[8] G. Zhang, Y. Wang, X. Wang, Y. Chen, Y. Zhou, Y. Tang, L. Lu, J. Bao, T. Lu, *Applied Catalysis B: Environmental* **2011**, 102, 614.

[9] K. Miao, Y. Luo, J. Zou, J. Yang, F. Zhang, L. Huang, J. Huang, X. Kang, S. Chen, *Electrochimica Acta* **2017**, 251, 588.

[10] B. Liu, H. Li, L. Die, X. Zhang, Z. Fan, J. Chen, *Journal of Power Sources* **2009**, 186, 62.

[11] H. Huang, A. Ruditskiy, S.-I. Choi, L. Zhang, J. Liu, Z. Ye, Y. Xia, *ACS applied materials & interfaces* **2017**, 9, 31203.

[12] P. Kannan, J. Dolinska, T. Maiyalagan, M. Opallo, *Nanoscale* **2014**, 6, 11169.

[13] J. Bai, L. Shen, D. Sun, Y. Tang, T. Lu, *CrystEngComm* **2014**, 16, 10445.

[14] S. Hu, F. Che, B. Khorasani, M. Jeon, C. W. Yoon, J.-S. McEwen, L. Scudiero, S. Ha, *Applied Catalysis B: Environmental* **2019**, 254, 685.

[15] T. T. Cheng, E. L. Gyenge, *Journal of applied electrochemistry* **2009**, 39, 1925.

[16] F. Saleem, Z. Zhang, B. Xu, X. Xu, P. He, X. Wang, *Journal of the American Chemical Society* **2013**, 135, 18304.

[17] L. Wei, Y.-J. Mao, F. Liu, T. Sheng, Y.-S. Wei, J.-W. Li, Y.-J. Fan, X.-S. Zhao, *ACS Applied Energy Materials* **2019**, 2, 7204.

[18] M. Zamanzad Ghavidel, E. B. Easton, *Catalysts* **2015**, 5, 1016.

[19] M. B. Askari, H. Beitollahi, A. Di Bartolomeo, *Nanomaterials* **2023**, 13, 679.

[20] S. Azizi, M. B. Askari, M. T. T. Moghadam, M. Seifi, A. Di Bartolomeo, *Nano Futures* **2023**, 7, 015002.

[21] R. Jana, U. Subbarao, S. C. Peter, *Journal of Power Sources* **2016**, 301, 160.

[22] H. Mao, T. Huang, A. Yu, *Electrochimica Acta* **2015**, 174, 1.

[23] A. Serov, T. Asset, M. Padilla, I. Matanovic, U. Martinez, A. Roy, K. Artyushkova, M. Chatenet, F. Maillard, D. Bayer, *Applied Catalysis B: Environmental* **2016**, 191, 76.

[24] P. Qiu, S. Lian, G. Yang, S. Yang, *Nano Research* **2017**, 10, 1064.

[25] J. Qi, N. Benipal, C. Liang, W. Li, *Applied Catalysis B: Environmental* **2016**, 199, 494.

[26] B. Y. Xia, W. T. Ng, H. B. Wu, X. Wang, X. W. Lou, *Angewandte Chemie* **2012**, 124, 7325.

[27] H. Zhang, Y. Yin, Y. Hu, C. Li, P. Wu, S. Wei, C. Cai, *The Journal of Physical Chemistry C* **2010**, 114, 11861.

[28] W.-k. Suh, P. Ganesan, B. Son, H. Kim, S. Shanmugam, *international journal of hydrogen energy* **2016**, 41, 12983.

[29] B. Ruiz-Camacho, J. Palafox-Segoviano, P. Pérez-Díaz, A. Medina-Ramírez, *International Journal of Hydrogen Energy* **2021**, 46, 26027.

[30] S. H. Park, C. H. Choi, J. K. Koh, C. Pak, S.-a. Jin, S. I. Woo, *ACS Combinatorial Science* **2013**, 15, 572.

[31] J. M. Linge, D. Kozhemyakin, H. Erikson, S. Vlassov, N. Kongi, K. Tammeveski, *ChemCatChem* **2021**, 13, 4364.

[32] S. Chakrabarty, A. Mukherjee, W.-N. Su, S. Basu, *International Journal of Hydrogen Energy* **2019**, 44, 1565.

[33] H. Erikson, M. Lüsi, A. Sarapuu, K. Tammeveski, J. Solla-Gullón, J. M. Feliu, *Electrochimica Acta* **2016**, 188, 301.

[34] Y.-L. Tsai, K.-L. Huang, C.-C. Yang, J.-S. Ye, L.-S. Pan, C.-L. Lee, *International journal of hydrogen energy* **2014**, 39, 5528.

[35] M. Zysler, V. Shokhen, S. S. Hardisty, A. Muzikansky, D. Zitoun, *ACS Applied Energy Materials* **2022**, 5, 4212.

[36] V. V. Doan-Nguyen, S. Zhang, E. B. Trigg, R. Agarwal, J. Li, D. Su, K. I. Winey, C. B. Murray, *ACS nano* **2015**, 9, 8108.
